# Effect of Chicken Egg Yolk Antibodies (IgY) against Diarrhea in Domesticated Animals: A Systematic Review and Meta-Analysis

**DOI:** 10.1371/journal.pone.0097716

**Published:** 2014-05-20

**Authors:** Thirumalai Diraviyam, Bin Zhao, Yuan Wang, Ruediger Schade, Antonysamy Michael, Xiaoying Zhang

**Affiliations:** 1 College of Veterinary Medicine, Northwest A&F University, Yangling, Shaanxi, China; 2 College of Science, Northwest A&F University, Yangling, Shaanxi, China; 3 Institute of Pharmacology, Charité - Universitätsmedizin Berlin, Berlin, Germany; 4 PSG College of Arts and Science, Bharathiar University, Coimbatore, Tamil Nadu, India; Institut National de la Santé et de la Recherche Médicale U 872, France

## Abstract

**Background:**

IgY antibodies are serum immunoglobulin in birds, reptiles and amphibians, and are transferred from serum to egg yolk to confer passive immunity to their embryos and offspring. Currently, the oral passive immunization using chicken IgY has been focused as an alternative to antibiotics for the treatment and control of diarrhea in animals and humans. This systematic review was focused to determine the effect of IgY in controlling and preventing diarrhea in domesticated animals including Piglets, Mice, Poultry and Calves.

**Methods and Results:**

Previous research reports focused on treatment effect of Chicken IgY against diarrhea were retrieved from different electronic data bases (MEDLINE, EMBASE, SPRINGER-LINK, WILEY, AGRICOLA, MEDWELL Journals, Scientific Publish, Chinese articles from Core periodicals in 2012). A total of 61 studies in 4 different animal classes met the inclusion criteria. Data on study characteristics and outcome measures were extracted. The pooled relative risk (RR) of 49 studies of different animals [Piglets – 22; Mice – 14; Poultry – 7 and Calves – 6] in meta-analyses revealed that, IgY significantly reduced the risk of diarrhea in treatment group when compare to the placebo. However, the 95% confidence intervals of the majority of studies in animal class piglets and calves embrace RR of one. The same results were obtained in sub group analyses (treatment regiment – prophylactic or therapeutic; pathogen type – bacterial or viral). Perhaps, this inconsistency in the effect of IgY at the individual study level and overall effect measures could be influenced by the methodological heterogeneity.

**Conclusion:**

The present systematic review (SR) and meta-analysis demonstrated the beneficial effect of IgY. This supports the opinion that IgY is useful for prophylaxis and treatment. However, more intensive studies using the gold standard animal experiments with the focus to use IgY alone or in combination with other alternative strategies are indispensable.

## Introduction

In a common way antibiotics have been used in animal agriculture for growth promotion (sub-therapeutic doses), disease prevention (prophylactic doses) and for the treatment (therapeutic dose) for more than 50 years and many of the research reports and practical experiences have shown the usage of antibiotic to significantly contribute for the improved performance of animals [1], [2]. However, the use and misuse of in-feed antibiotics have resulted in serious complications due to drug residues in animal products and increased bacterial resistance. The recognition of these dangers prompted the ban on sub therapeutic usage of antibiotics in many developed countries and some of the developing countries are seriously considering a similar ban. Therefore a viable alternative strategy to antibiotics is essentially needed to combat drug-resistance microorganisms and also to treat the diseases that are unresponsive to drug therapy (viral infection) and for individuals with impaired immune systems who are unable to respond to conventional vaccines [3].

A broad range of products as an effective alternative to antibiotics have been focused seriously by the global biomedical research community. Recently, passive immunization using chicken egg yolk immunoglobulin (IgY) has become an attractive approach with considerable attention as it possesses a variety of advantages over mammalian IgG such as convenience, high yield and cost-effectiveness. Oral administration of specific chicken IgY has been shown to be effective against a variety of intestinal pathogens especially diarrheal pathogens in different animal classes and humans such as bovine and human rotaviruses, bovine coronavirus, enterotoxigenic *Escherichia coli* (ETEC) and *Salmonella* spp. [4]. Although the beneficial effects of chicken IgY in controlling or preventing the diarrheal disease in animals have been known for more than two decades and reported by many researchers globally, it still remains a difficult task to use chicken IgY as an alternative to conventional treatment with strong scientific conclusion. At this juncture, a meta-analysis is necessary and helpful to summarize the previous research findings and provide a comprehensive conclusion and proper direction for further research. The objective of this SR and meta-analysis is to determine the effect of chicken IgY for the treatment and control of diarrhea in domesticated animals using available research reports on IgY against diarrhea in different animal classes like piglets, mice, poultry and claves.

## Materials and Methods

The review protocol is publicly available at CAMARADES (Collaborative Approach to Meta Analysis and Review of Animal Data from Experimental Studies) web site (http://www.camarades.info/index_files/chickenyolkSRt.pdf).

### 1. Literature search

Previous research reports were retrieved from different electronic data bases including MEDLINE, EMBASE, SPRINGER-LINK, WILEY, AGRICOLA, MEDWELL Journals, Scientific Research Publish and Chinese articles from Core periodicals in 2012 regardless of language and publication status. The search strategies are presented in our review protocol available at CAMARADE as well as in supporting information. Bibliographies of narrative review articles and eligible trials were reviewed manually for the potential studies which cannot be retrieved by the electronic searches. All these literature searches were carried out until September 30, 2013. Besides, the publications in the conference proceedings, dissertation abstracts and other studies that would have to be in “file drawers” (unpublished manuscripts) were also considered for the analyses in order to reduce the risk of missing potential studies.

### 2. Selection of potential studies

The research studies reported on the effect of IgY antibodies as a treatment agent for controlling and preventing diarrhea in animals were selected on the basis of the title and abstract by 2 independent authors (TD and XYZ). Full articles were reviewed when a decision could not be made using abstract. 2 reviewers independently evaluated the eligibility of each potential full-text article and resolved the disagreement by consensus and discussion with the third party (RS and AM).

### 3. Inclusion criteria

**Figure 1:**
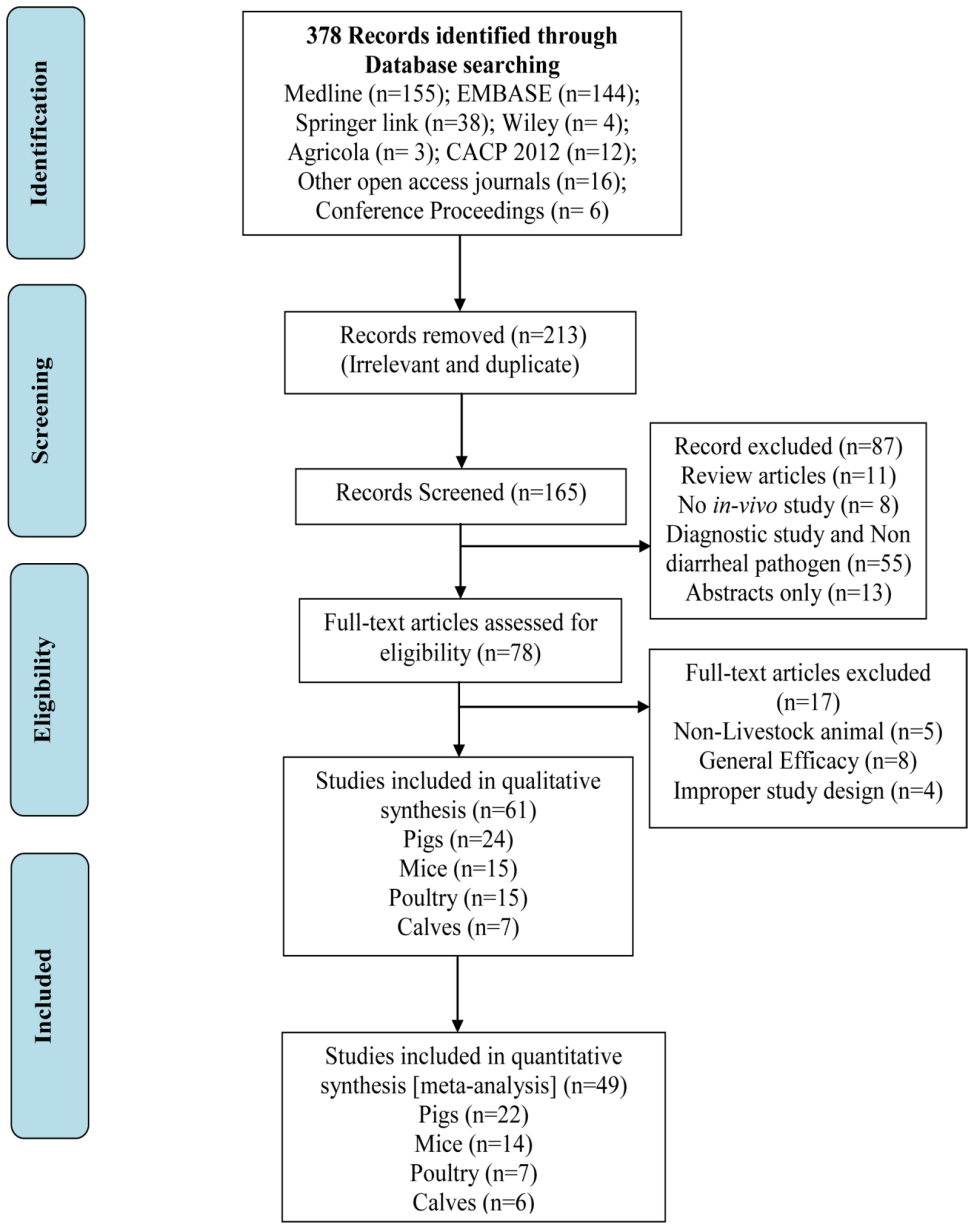
Summary of literature search, screening and selection. PRISMA flow diagram represents the literature search in different electronic data base followed by screening and inclusion of eligible studies for systematic review and meta-analysis.

Research reports that were published in English, Chinese and German were selected and included for the analyses, having met the following inclusion criteria: 1) Study with in-vivo animal experiment [should have been conducted with animal ethical committee approval] 2) Study should have evaluated the effect of IgY against diarrhea causing pathogens 3) Study should have used any one formulation of IgY: purified IgY, partially purified IgY, immune yolk (liquid) or immune yolk powder (spray dried or freeze dried) 4) The treatment effect of IgY was assessed using one of the following outcomes: mortality rate, survival rate, fecal score for pathogen excretion, fecal consistency (diarrhea) and body weight 5) Sufficient data should be described including number of animals in each group (treatment and control), pathogen challenge dose and IgY treatment dose with duration, hypothetical consent either prophylactic or therapeutic effect of IgY. PRISMA flow diagram for literature selection, screening and inclusion has been presented in Figure 1 [5].

### 4. Data extraction

Studies included for qualitative and quantitative analysis were critically assessed by three independent reviewers (TD, XYZ and YW) and the following data were extracted: year of publication with first author, information pertaining to the hypothesis of IgY usage (therapeutic or prophylactic), animals class, breed, age, body weight, type of infection (experimental or natural-field trail), pathogen (bacterial or viral), strain, challenge dose and time, dose of IgY, mode of administration, duration of the treatment, number of animals in control and treatment group, outcome assessment (Table S1, S2, S3 and Table 1). Missing information was discussed with the corresponding authors by contacting through e-mail or telephonic conversation. Reviewers assessed the risk of bias, inconsistency and indirectness of the study included for the systematic review.

**Table 1:** Characteristics of the included study – Calves.

Author & Year	Experimental Animal	Infection Dose	IgY Treatment	Outcome Assessment (Type of Efficacy)
**Animal Class: Calves**				
**Bacterial Pathogen**				
**Ozpinar et al., 1996**	New born Calves of Holstein Frisian breed and brown cattel	Field Trail: Routine diagnosis in the farm on day 7-In the diarrheic calves 78.2% for rotavirus, 34.5% for Cryptosporidia, 18% for *E. coli* and 42% for polyinfection	Field Trial: 2 g, 4 g, or 8 g egg powder with specific antibodies to rotavirus types 1 and 2 [Neutralization titer512] and *E.coli* K99 pilus antigen [ELISA titer 330] each, in a drink – two meal/day for the first 14 days	Examined the incidence of diarrhea, duration of diarrhea, weight gain and mortality (P)
**Cook et al., 2005**	Canadian Arcott rams [Sheep] [38.8 kg]	10^10^ CFU of three strain mixture of *E.coli* O157:H7/sheep on day 0 using 60-mL syringe connected to a polypropylene orogastric tube	100 g of Spray dried egg yolk powder suspended in a final volume of 300 mL PBS–on day 2, 3 and 4 by syringe and orogastric tube. Treatments: 1) 100 g non-immunized egg powder [EG]; 2) 100 g - immunized egg powder [High]; 3) 100 g [50 g EG+50 g immunized egg] [Medium]; 4) 100 g [75 g EG+25 g immunized egg [Low]; 5) variable doses consisting of High on day 2, Medium on day 3 and Low on day 4	Fecal shedding of *E.coli* O157:H7 in five consecutive weekly samples (T)
**Germine ** ***et al*** **., 2011**	New born Calves of non-vaccinated dams and deprived from colostral antibodies- below 30 days old	Field trail – against *E. coli* K99	20 mL yolk/calf – mixed with 1.5–2.0 kg of milk 2 times/day for 21days – then calves fed only milk only according to their weight	Examined the *E. coli* K99 in the faecal samples (P)
**Viral Pathogen**				
**Kuroki et al., 1994**	Neonatal Holstein Calves	Shimane BRV - 1×10^10^ TCID_50_/Calf (Gp1-3) KK-3 BRV −5×10^9^ TCID_50_/Calf (Gp1-6) The challenge time was 2 hours after first dose of IgY on 2 day after birth	Gp1 and Gp4 control IgY Gp 2 and Gp 3 received anti-Shimane IgY [3200 and 6400 titer] Gp 5 and Gp 6 received anti-KK-3 IgY [6400 and 12800 titer] – delivering the solution via syringe before giving milk formula ration	Fecal score, viral excretion in feces and body weight gain observed (C)
**Ikemori et al., 1997**	Colostrum deprived, newborn Holstein Calves	1×10^9.0^ TCID_50_ of the Kakegawa strain of BCV (at 24 to 36 h from birth)	Egg powder groups: 0.25 g [1∶1280titer] and 0.5 g [1∶2560] in 1.5 and 2 Liters of milk – 2times/day for 7 days after challenge (6 h after challenge)	Evaluated fecal consistency score, weight gain, and mortality (T)
**Kuroki et al., 1997**	Japanese black Neonatal Calves	Three field trails – against BRV	2 g of combined anti-Shimane and KK-3 IgY [each with homotypic titer of 12800] 3times/day for 2 weeks after birth in 50 mL distilled water – oral delivery by50 mL syringe	Fecal score and body weight gain were examined (P)
**Vega et al., 2011**	New born Holstein male Calves removed prior to suckling within the first 4 h of life	10^5.85^ FFU of virulent INDIANA BRV between 3^rd^ & 4th feeding [36 h after colostrums intake; 0 post inoculation day]	GP1: Control Colostrum (CC) + milk with BRV-specific egg yolk with a final titer of 4096; Gp2: CC+ milk with normal egg yolk Gp3: only one dose of CC Gp4: Colostrum deprived (Gp1 and 2 – received 2 L of Antibody supplemented milk 2 times/day for 14days)	Examined for diarrhea and virus shedding with advanced immunological assays (P)

**Legend**: CFU colony forming unit, TCID Tissue culture infective dose, FFU Focus forming unit, BRV Bovine Rotavirus, BCV Bovine Coronavirus, Type of Efficacy: P-Prophylactic Effect; T-Therapeutic Effect; F-Field Trial.

### 5. Assessing the quality of the studies

Potential studies were assessed for methodological quality using the Animal Research: Reporting of In-vivo Experiment (ARRIVE) Guidelines developed as a part of NC3Rs (National Centre for the Replacement, Refinement and Reduction of Animals in Research) to improve the design, analysis and reporting of research using animals [6], [7]. The quality was assessed by evaluating whether the methodology of included studies met the ARRIVE guideline or not. Maximum score of 20 points was attributed to each article. Based on the total score, studies were categorized in to three different groups as good, moderate and poor if the score percentage was ≥75%, ≥65% and ≤55% respectively (Table 2).

**Table 2:** Quality of Animal Studies: Values are numbers (percentage).

Animal Class (Number of Studies)	Quality of Animal Studies as per ARRIVE Guidelines*		
	Good (≥75%)	Moderate (≥65%)	Poor (≤55%)
**Pigs (n = 24)**	12 (50)	9 (37.5)	3(12.5)
**Mice (n = 15)**	11 (73)	4 (27)	-
**Poultry (n = 15)**	7 (46.6)	5 (33.4)	3 (20)
**Ruminant (n = 7)**	2 (28.5)	3 (43)	2 (28.)
**Total**	**32 (52.5)**	**21(34.4)**	**8 (13.1)**

*ARRIVE Guidelines– Animal Research: Report of In-vivo experiment Guideline.

**Figure 2:**
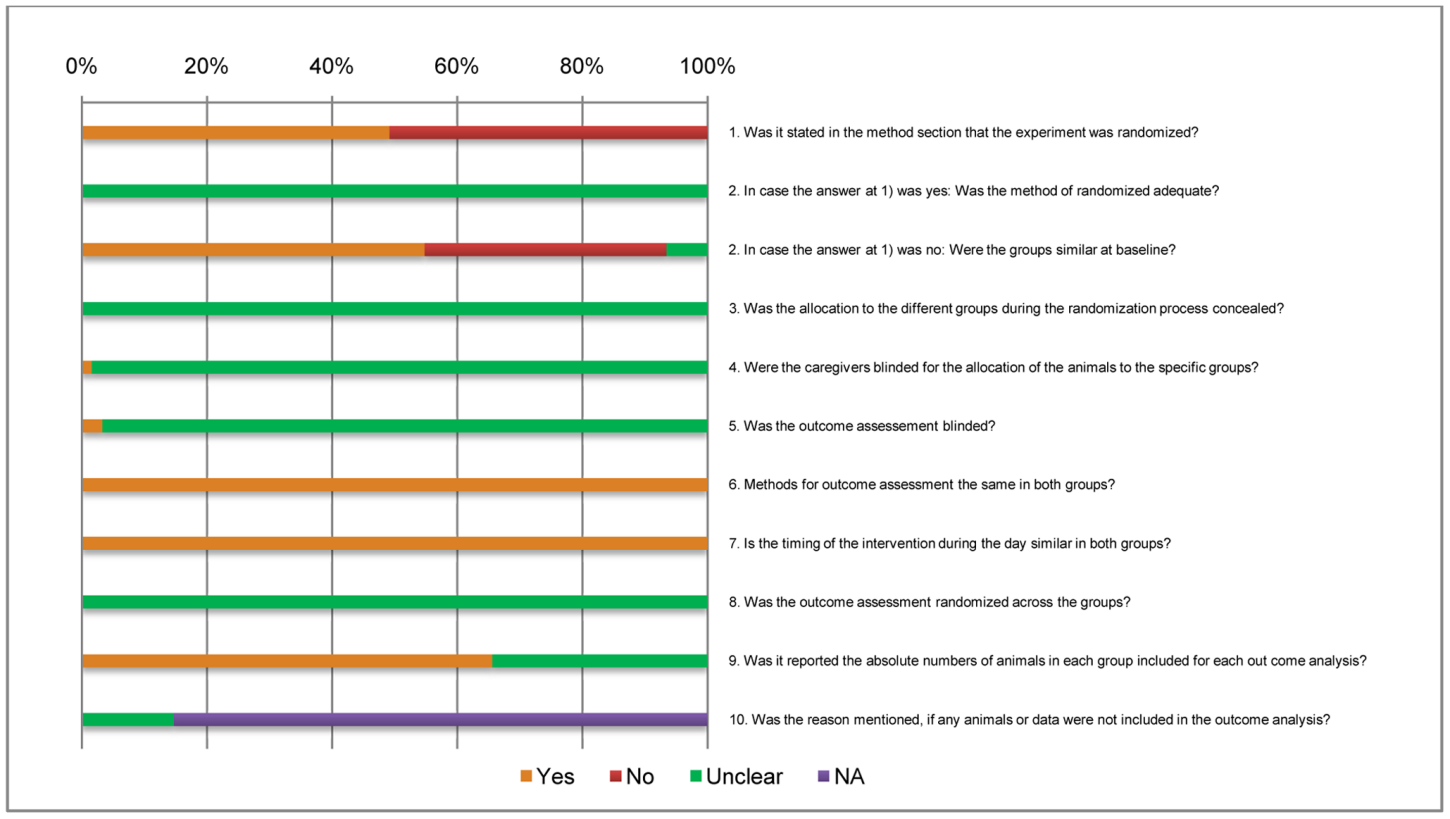
Risk of bias assessment. Yes = low risk of bias, No = high risk of bias, Unclear = unclear risk of bias, NA = not applicable.

Risk of bias of the included studies was measured by two reviewers (TD and X-YZ) using the criteria designed already with slight modification (in items 9 and 10) such as the possible presence of selection bias (items 1, 2 and 3), performance bias (items 4 and 7), detection bias (items 5, 6 and 8) and attrition bias (items 9 and 10) [8], [9]. The scores such as “Yes” denotes low risk of bias, “No” denotes high risk of bias, “Unclear” denotes unknown risk of bias, “NA” indicates non applicable (Figure 2).

### 6. Data synthesis and statistical analysis

In accordance to the differences between studies such as different doses, formulation of IgY, pathogenic strains used for challenge, duration of treatment, outcome assessment and age of animals; the random-effects meta-analysis was taken in to consideration for calculating the relative risk (RR) and the 95% confidence interval (CI) in trails reporting the binary outcome i.e., number of animals with diarrhea or number of animals died by diarrhea (mortality rate). Since, the number of studies in piglets are relatively higher than other animal class, the subgroup analysis were carried out for the studies reported only in Piglets with following study characteristics, pathogen type – bacterial or viral and treatment regiment – prophylactic or therapeutic. In order to estimate the amount of heterogeneity, Q and *I* ^2^ value was calculated [10]. The presence of publication bias was assessed by funnel plots and Egger regression test [11]. Meta-analysis was performed by MedCalc Statistical Software version 12.7.7 (MedCalc Software bvba, Ostend, Belgium; 2013) [12]. Forest plot graphical representation was used to display the meta-analysis results (BZ). Other study characteristics and results were summarized narratively with tables and running text.

## Results

### 1. Characteristics of the included studies

A total of 378 records were identified through different database searching: Medline (n = 155); EMBASE (n = 144); Springer link (n = 38), Chinese articles from Core periodicals in 2012 (n = 12); Wiley (n = 4); Agricola (n = 3); other open access journals (n = 16) and conference proceedings (n = 6). After intensive screening and eligibility to met inclusion criteria a total of 61 studies [Piglets – 24; Mice – 15; Poultry – 15 and Calves – 7] [13]–[73] were included for systematic review, of which 49 records [Piglets – 22; Mice – 14; Poultry – 7 and Calves – 6] were included for meta-analysis (Figure 1). Chinese and German articles were translated by three reviewers (XYZ, YW and RS). The characteristics of each included studies are documented and presented in Table S1, S2, S3 and Table 1.

The study characteristics had considerable difference among the records included for analyses; in particular IgY dose and formulation are not comparatively homologous. Some of the studies mentioned, that IgY administration started before challenging with pathogen (prophylactic effect) whereas in some trials it was after the onset of diarrhea (therapeutic effect). Few studies have reported that IgY administration had begun immediately after challenging. In terms of diarrhea, 12 experiments among different studies were performed to evaluate the effect of IgY in the field (farm) whereas other studies reported the effect of IgY against the experimental diarrhea. In poultry trails, some studies focused to assess the effect of IgY to prevent the possible bacterial contamination (pathogens responsible for diarrhea in animals and humans) in the eggs.

### 2. Risk of bias and quality of studies

The quality of the included studies as per ARRIVE Guidelines: individual study analysis revealed that none of the studies showed 100% quality, barely very few studies scored more than 90%. In categorization, 52.5% of the studies are good, 34.4% studies are moderate and 13.1% studies are poor. Which indicates that, the nearly 50% of the included studies are relatively low in quality (Table 2). The overall result for the risk of bias assessment for the 61 studies included was presented in Figure 2. The results indicated 49.2% studies reported that experimental groups of animals were randomly assigned, but there was not description regarding the method of randomization. None of the studies discussed about the grouping concealment. Only three of these studies described about the blinded outcome assessment and only one of which provided the complete details. Out of the 10 items, only two items 7 and 8 show the low risk of bias for all the studies and many items are scored as “unclear risk of bias”. These findings clearly state the inadequacy of quality and standard in the reporting of animal studies on effect of IgY against diarrhea in different animal classes. In addition, the existence of these biases in animal experiments possibly influences the outcome of this systematic review and meta-analysis.

### 3. Effect of chicken IgY against diarrhea in animals

Studies on passive immunization by oral administration of IgY for the treatment and control of diarrhea in different animal classes such as piglets, mice, poultry and calves were selected and statistically analyzed to have comprehensive evidence regarding the effect of IgY. The following results were found for each animal class.

#### 3.1. Effect of IgY against diarrhea in Piglets

**Figure 3:**
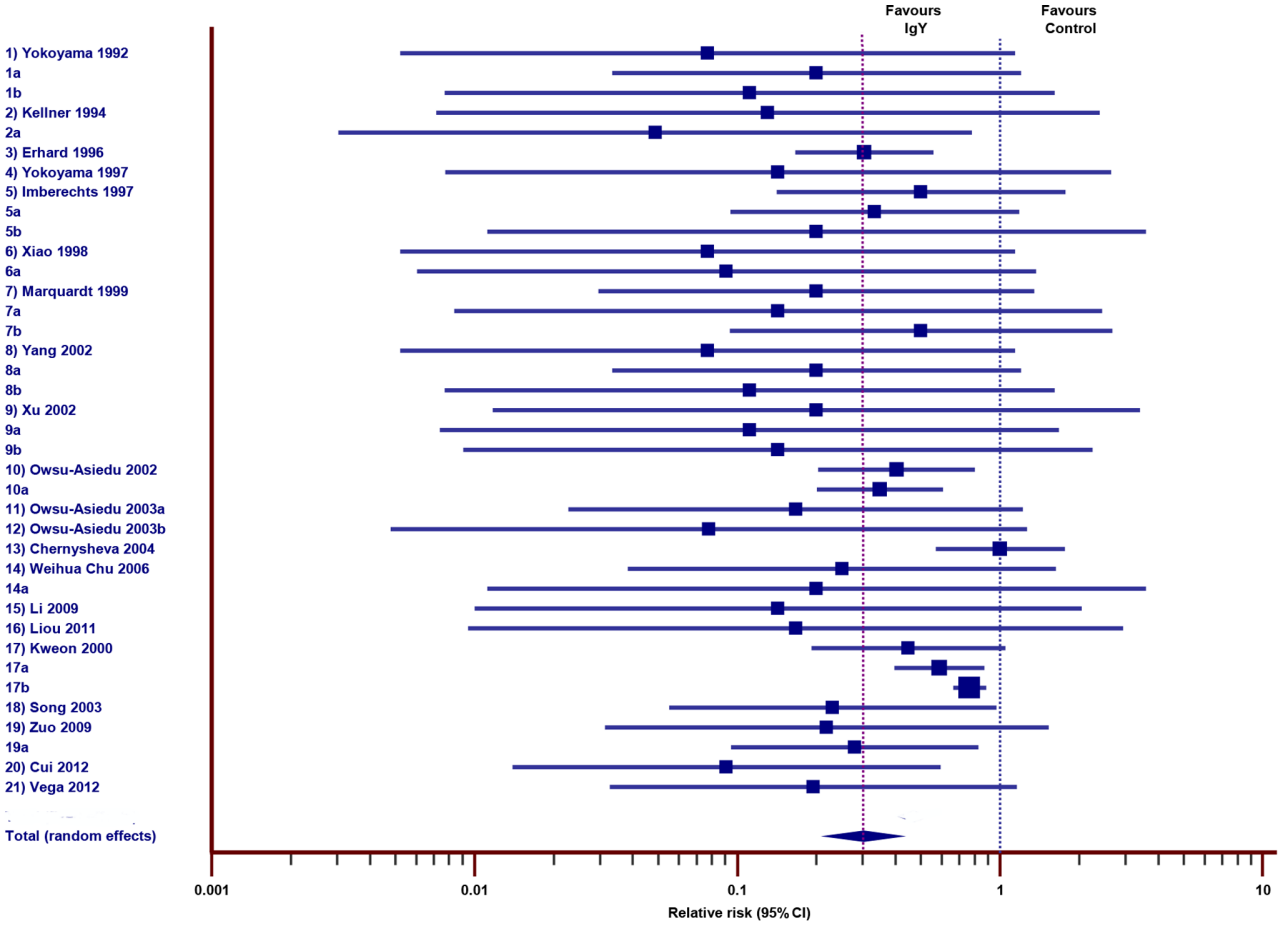
Effect of IgY against diarrhea in piglets. Forest plot demonstrates the relative risk (RR) of individual studies included for meta-analysis under animal class piglets, 95% confidence interval. The diamond represents the global estimate and its 95% confidence interval. The cut off line crossing RR 1 differentiates the study favors IgY treatment group or control group. The line crossing diamond is to determine the number of studies positioned in global RR.

Out of 61studies selected for analysis, 24 studies were reported on IgY effect against diarrhea in piglets, in which 22 studies were included for meta-analysis including 17 studies against bacterial diarrhea and 5 studies against viral diarrhea (a total of 39 experiments). Meta-analysis indicated, that majority of the studies (except 8 experiments) overlaps the RR of 1at 95% confidence interval, even though the overall effect estimate was statically significant (Figure 3 and Table 3; pooled RR,0.30; 95% CI 0.22 to 0.41). The heterogeneity was moderate (Q = 86.63; DF = 37; P<0.0001, I^2^ = 57.29%). Studies of Zuniga et al 1997 and Girard et al 2006 were not included in the meta-analysis, since they reported the continuous variables such as amount of viable cells in the feces (log colony forming unit g^−1^) and reduction in the mean percentage of inter-crypt epithelium (ICME); but both the studies supported the oral application of egg yolk antibodies for prevention of infectious diseases caused by enteric pathogens.

**Table 3:** Effect of IgY against diarrhea in Piglets.

Study	No. of mortality by diarrhea (or) No. with diarrhea/No. in Group (%)		Outcome measure considered - Mortality (M) or Diarrhea (D)	Relative Risk (RR)	95% Confidence Interval (CI)
	Intervention	Control			
**Animal Class: Pig**					
**Bacterial Pathogen**					
Yokoyama 1992	0/7 (0)	6/7 (86)	M	0.077	0.00521 to 1.136
	0/4 (0)	4/4 (100)	M	0.111	0.00814 to 1.516
	0/5 (0)	4/5 (80)	M	0.111	0.00768 to 1.608
Kellner 1994	0/23 (0)	3/21 (14.3)	D	0.131	0.00716 to 2.387
	0/18 (0)	7/13 (58.3)	D	0.049	0.00303 to 0.781
Erhard 1996	10/58 (17.2)	34/60 (56.7)	D	0.304	0.166 to 0.558
Yokoyama 1997	0/28 (0)	3/28 (11)	D	0.143	0.00772 to 2.642
Imberechts 1997	2/6 (33)	4/6 (66)	D	0.500	0.141 to 1.772
	2/8 (25)	6/8 (75)	D	0.333	0.0941 to 1.181
	0/8 (0)	2/8 (25)	M	0.200	0.0112 to 3.576
Xiao 1998	0/7 (0)	6/7 (85.7)	M	0.077	0.00521 to 1.136
	0/7 (0)	5/7 (71.4)	M	0.091	0.00603 to 1.370
Marquardt 1999	1/8(12.5)	5/8(26.5)	M	0.200	0.0296 to 1.351
	0/10(0)	3/10 (30)	M	0.143	0.00837 to 2.438
	2/102(1.9)	4/102 (3.9)	D	0.500	0.0936 to 2.670
Yang 2002	0/7 (0)	6/7 (85.7)	M	0.077	0.00521 to 1.136
	0/4 (0)	4/4 (100)	M	0.111	0.00814 to 1.516
	0/5 (0)	4/5 (80)	M	0.111	0.00768 to 1.608
Xu 2002	0/6 (0)	2/6 (33.3)	M	0.200	0.0117 to 3.406
	0/6 (0)	4/6 (66.7)	M	0.111	0.00738 to 1.673
	0/6 (0)	3/6 (50)	M	0.143	0.00907 to 2.249
Owsu-Asiedu 2002	7/24(30)	13/18 (73)	D	0.404	0.203 to 0.802
	8/24(33)	18/18 (100)	D	0.333	0.189 to 0.587
Owsu-Asiedu 2003a	1/15(6.6)	6/15 (40)	M	0.167	0.0227 to 1.222
Owsu-Asiedu 2003b	0/18 (0)	8/24(33)	M	0.078	0.00480 to 1.265
Chernysheva 2004	8/12 (66)	8/12 (66)	D	1.000	0.568 to 1.761
Weihua Chu 2006	1/6 (16.7)	4/6 (66.7)	M	0.250	0.0383 to 1.633
	0/8 (0)	2/8 (25)	M	0.200	0.0112 to 3.576
Li 2009	0/4(0)	3/4 (75)	D	0.143	0.01000 to 2.041
Liou 2011	0/10 (0)	2.5/10 (25)	M	0.167	0.00946 to 2.937
**Viral Pathogen**					
Kweon 2000	5/19 (26)	10/17(58)	M	0.447	0.191 to 1.048
	18/43 (41)	35/49 (71)	M	0.586	0.395 to 0.869
	201/396 (50)	118/178 (66)	M	0.766	0.664 to 0.883
Song 2003	1/6 (16.7)	6/6 (100)	M	0.167	0.0278 to 0.997
Zuo 2009	1/8 (12.5)	4/7 (57)	M	0.219	0.0314 to 1.526
	3/17 (17)	12/19 (63)	M	0.279	0.0946 to 0.825
Cui 2012	0/10 (0)	10/10 (100)	M	0.048	0.00318 to 0.712
Vega 2012	0/4(0)	6/6(100)	D	0.111	0.00814 to 1.516
Pooled random effects				0.302	0.221 to 0.413

**Test for Heterogeneity**: Q = 86.63; DF = 37; P<0.0001, I^2^ = 57.29% (95% CI for I^2^ = 38.72 to 70.23).

#### 3.2. Effect of IgY against diarrhea in Mice

**Figure 4:**
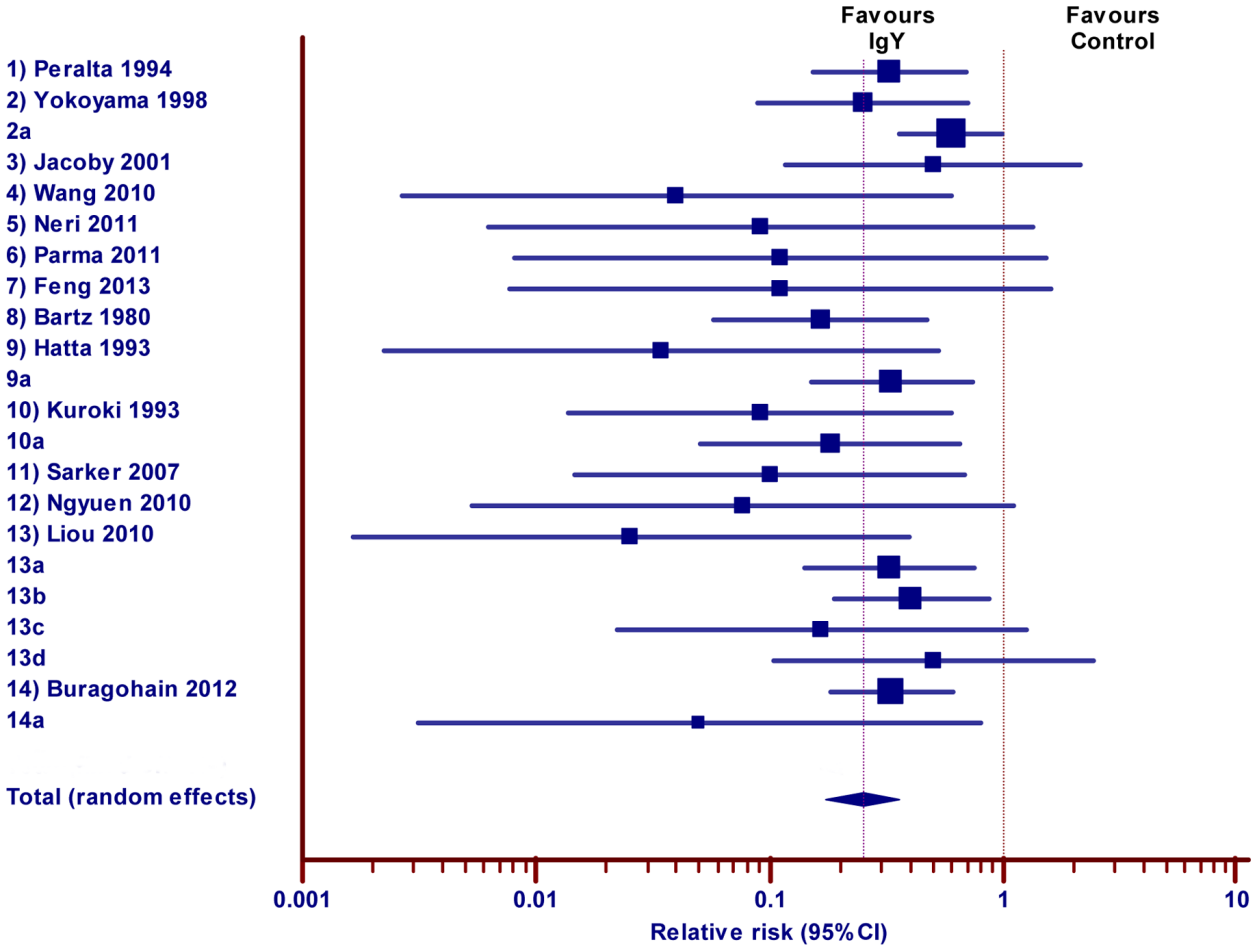
Effect of IgY against diarrhea in mice. Forest plot demonstrates the relative risk (RR) of individual studies included for meta-analysis under animal class mice, 95% confidence interval. The diamond represents the global estimate and its 95% confidence interval. The cut off line crossing RR 1 differentiates the study favors IgY treatment group or control group. The line crossing diamond is to determine the number of studies positioned in global RR.

Fifteen records were identified those reported on the effect of IgY against diarrhea in Mice and 14 studies (22 experimental trails) were included for meta-analysis. Results revealed the statistically significant effect of IgY for reducing the risk of diarrhea in intervention group treated with specific IgY compared with the control group at the individual study level (16 experiments out of 22 trials reported in 14 studies) as well as the meta-analysis level (Figure 4 and Table 4; pooled RR, 0.25; 95% CI 0.18 to 0.36). The low level of heterogeneity (Q = 36.17; DF = 21; P = 0.0209, I^2^ = 41.94%) was observed among the studies. Report of Hirai et al 2010 was not included in the meta-analysis due to lack of description for absolute number of experimental animals (results were presented as percentage of survival), the study demonstrated, that the mixture of anti-*Vibrio cholera* and anti-cholera toxin subunit-B IgYs could be used to prevent or treat cholera caused by either O1 or O139.

**Table 4:** Effect of IgY against diarrhea in Mice.

Study	No. of mortality by diarrhea (or) No. with diarrhea/No. in Group (%)		Outcome measure considered - Mortality (M) or Diarrhea (D)	Relative Risk (RR)	95% Confidence Interval (CI)
	Intervention	Control			
**Animal Class: Mice**					
**Bacterial Pathogen**					
Peralta 1994	6/27 (22)	17/25 (68)	M	0.327	0.154 to 0.695
Yokoyama 1998	3/15 (20)	12/15 (80)	M	0.250	0.0881 to 0.710
	6/10 (60)	10/10 (100)	M	0.600	0.362 to 0.995
Jacoby 2001	2/10 (20)	4/10 (40)	D	0.500	0.117 to 2.139
Wang 2010	0/12 (0)	12/12 (100)	M	0.040	0.00265 to 0.605
Neri 2011	0/6 (0)	5/6 (83)	M	0.091	0.00621 to 1.330
Parma 2011	0/4 (0)	4/4 (100)	M	0.111	0.00814 to 1.516
Feng 2013	0/5 (0)	4/5 (80)	M	0.111	0.00768 to 1.608
**Viral Pathogen**					
Bartz 1980	3/20 (15)	30/33 (91)	D	0.165	0.0578 to 0.471
Hatta 1993	0/16(0)	14/16(83)	D	0.035	0.00224 to 0.532
	4/12 (37)	12/12 (100)	D	0.333	0.150 to 0.742
Kuroki 1993	1/12 (8.33)	11/12 (91)	D	0.091	0.0138 to 0.598
	2/12 (16.6)	11/12 (91)	D	0.182	0.0507 to 0.652
Sarker 2007	1/15 (6)	10/15 (67)	D	0.100	0.0146 to 0.687
Ngyuen 2010	0/6 (0)	6/6 (100)	M	0.077	0.00536 to 1.103
Liou 2010	0/21 (0)	21/23 (91)	M	0.026	0.00164 to 0.395
	5/25 (20)	16/26 (62)	M	0.325	0.140 to 0.753
	5/16 (31)	17/22 (77)	M	0.404	0.189 to 0.866
	1/19 (5)	6/19 (32)	M	0.167	0.0221 to 1.255
	2/19 (11)	4/19 (21)	M	0.500	0.104 to 2.412
Buragohain 2012	7/21 (33)	23/23 (100)	D	0.333	0.182 to 0.610
	0/19 (0)	9/18 (50)	D	0.050	0.00312 to 0.798
**Pooled random effects**				0.250	0.175 to 0.358

**Test for Heterogeneity**: Q = 36.17; DF = 21; P = 0.0209, I^2^ = 41.94% (95% CI for I^2^ = 3.60 to 65.03).

#### 3.3. Effect of IgY against diarrhea in Poultry

**Figure 5:**
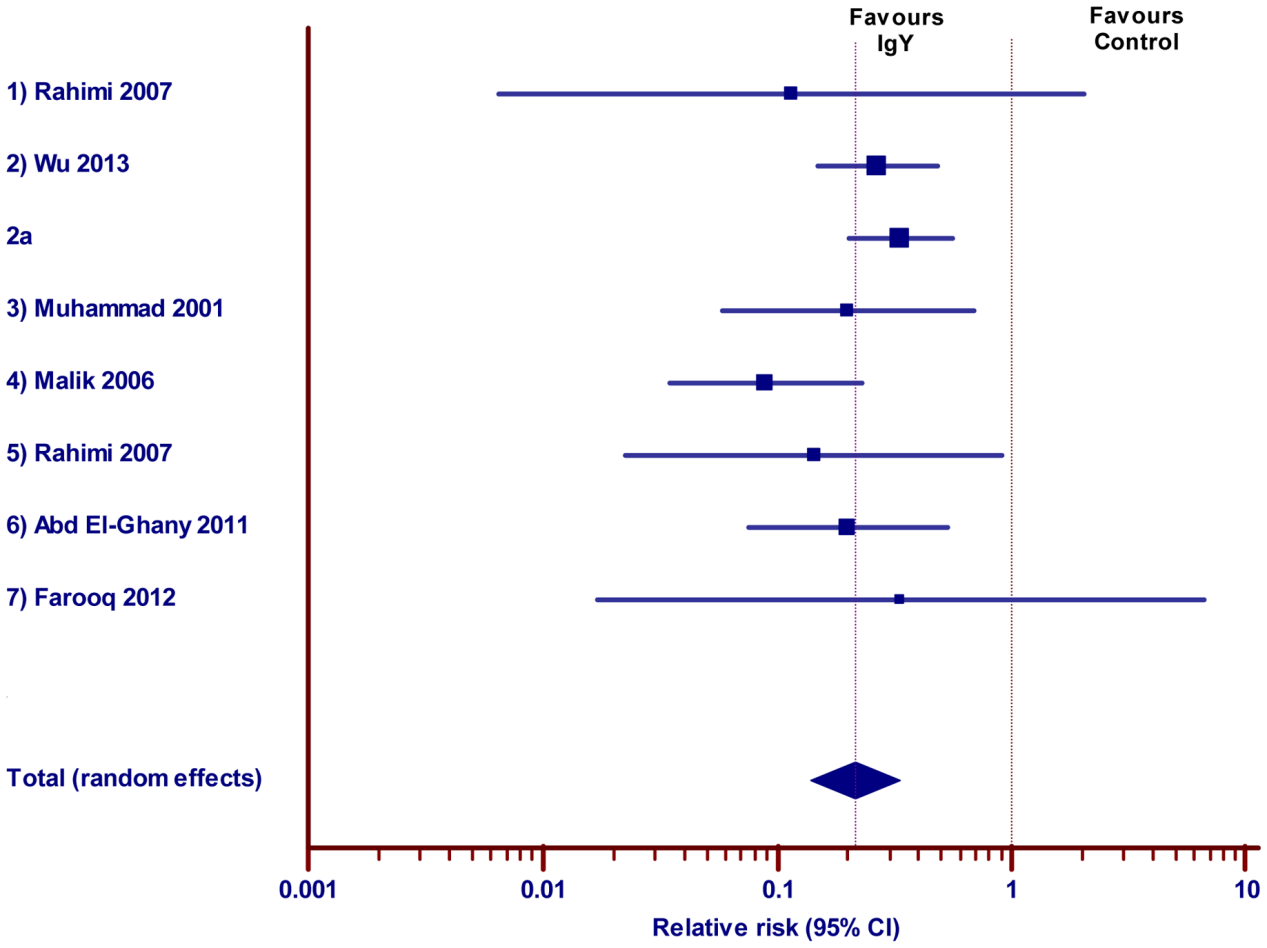
Effect of IgY against diarrhea in poultry. Forest plot demonstrates the relative risk (RR) of individual studies included for meta-analysis under animal class poultry, 95% confidence interval. The diamond represents the global estimate and its 95% confidence interval. The cut off line crossing RR 1 differentiates the study favors IgY treatment group or control group. The line crossing diamond is to determine the number of studies positioned in global RR.

A total of 15 studies demonstrating the effect of IgY against diarrheagenic pathogens in poultry were selected. Of which 8 (>50%) reports were excluded from meta-analysis, because 6 studies presented the outcome measures as graphical representations and statistical derivatives (not absolute number of birds); 2 records were reported the reduction in the possible bacterial contamination of eggs. The meta-analysis showed, that the administration of IgY reduces the risk of event significantly in the treated group when compare to the untreated control (Figure 5 and Table 5; pooled RR, 0.22; 95% CI 0.14 to 0.34). The beneficial effect of IgY could be further supported by the 95% confidence intervals at individual study level (6 experiments reported in the 5 studies) did not embrace a RR of 1. The heterogeneity was low between the included studies (Q = 10.14; DF = 7; P = 0.1807, I^2^ = 30.98%).

**Table 5:** Effect of IgY against diarrhea in Poultry.

Study	No. of mortality by diarrhea (or) No. with diarrhea/No. in Group (%)		Outcome measure considered - Mortality (M) or Diarrhea (D)	Relative Risk (RR)	95% Confidence Interval (CI)
	Intervention	Control			
**Animal Class: Poultry**					
**Bacterial Pathogen**					
Rahimi 2007	0/26 (0)	4/27 (14)	D	0.115	0.00652 to 2.039
Wu 2013	8/30 (26.6)	10/10 (100)	M	0.267	0.147 to 0.483
	10/30(33.3)	10/10 (100)	M	0.333	0.201 to 0.553
**Viral Pathogen**					
Muhammad 2001	2/10 (20)	10/10 (100)	M	0.200	0.0579 to 0.691
Malik 2006	4/50 (8)	45/50 (90)	M	0.089	0.0346 to 0.229
Rahimi 2007	1/8 (12.5)	7/8 (87.5)	D	0.143	0.0224 to 0.910
Abd El-Ghany 2011	4/40 (10)	20/40 (50)	M	0.200	0.0751 to 0.533
Farooq 2012	0/5 (0)	1/5 (20)	M	0.333	0.0170 to 6.526
**Pooled random effects**				**0.216**	**0.140 to 0.335**

**Test for Heterogeneity**: Q = 10.14; DF = 7; P = 0.1807, I^2^ = 30.98% (95% CI for I^2^ = 0.00 to 69.27).

#### 3.4. Effect of IgY against diarrhea in Calves

**Figure 6:**
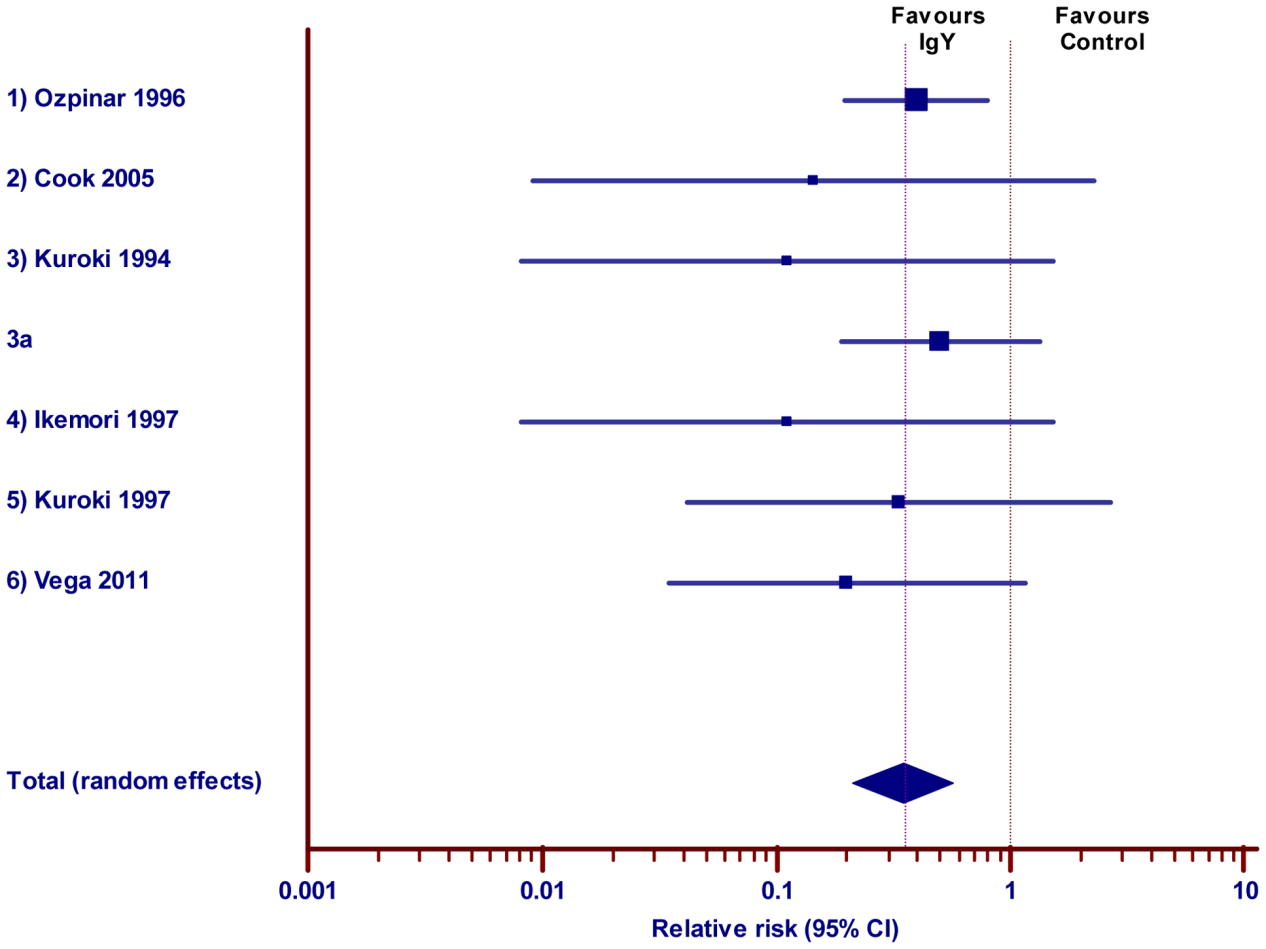
Effect of IgY against diarrhea in calves. Forest plot demonstrates the relative risk (RR) of individual studies included for meta-analysis under animal class Calves, 95% confidence interval. The diamond represents the global estimate and its 95% confidence interval. The cut off line crossing RR 1 differentiates the study favors IgY treatment group or control group. The line crossing diamond is to determine the number of studies positioned in global RR.

Six studies were included for meta-analysis out of 7 studies investigated the effect of IgY against diarrhea in calves. As mentioned in the previous animal class, one study was excluded due to insufficient information about the number of animals in the outcome measures. Most of the studies were performed as field trail; it might be the reason that rearing cost of cows to have calves for animal experiment, further the size of the animals is relatively lager. Results revealed, that the 95% confidence intervals of all the studies except one study overlap the RR of 1, hence there is no statistical significance at the individual study level. However, the overall effect estimate and its 95% confidence intervals indicated the beneficial effect of IgY (Figure 6 and Table 6; pooled RR, 0.35; 95% CI 0.21 to 0.57) with very low level of heterogeneity (Q = 3.13; DF = 6; P = 0.7924, I^2^ = 0.00%).

**Table 6:** Effect of IgY against diarrhea in Calves.

Study	No. of mortality by diarrhea (or) No. with diarrhea/No. in Group (%)		Outcome measure considered - Mortality (M) or Diarrhea (D)	Relative Risk (RR)	95% Confidence Interval (CI)
	Intervention	Control			
**Animal Class: Calves**					
**Bacterial Pathogen**					
Ozpinar 1996	8/54 (14)	30/80 (38)	D	0.395	0.196 to 0.795
Cook 2005	0/6 (0)	3/6 (50)	D	0.143	0.00907 to 2.249
**Viral Pathogen**					
Kuroki 1994	0/4 (0)	4/4 (100)	D	0.111	0.00814 to 1.516
	2/4 (50)	4/4 (100)	D	0.500	0.188 to 1.332
Ikemori 1997	0/4 (0)	4/4 (100)	M	0.111	0.00814 to 1.516
Kuroki 1997	1/10 (10)	3/10 (30)	M	0.333	0.0414 to 2.686
Vega 2011	1/5(20)	6/6 (100)	M	0.200	0.0346 to 1.154
**Pooled random effects**				**0.348**	**0.212 to 0.571**

**Test for Heterogeneity**: Q = 3.1297; DF = 6; P = 0.7924, I^2^ = 0.00% (95% CI for I^2^ = 0.00 to 44.96).

#### 3.5. Sub-group analysis

In order to perform the sub-group analysis, 24 piglets studies were considered, of which 16 studies were selected to evaluate the prophylactic effect of IgY and 14 studies were identified to determine the therapeutic effect of IgY (regardless of pathogen type). There was no statistical significance at individual study level but overall effect estimate did not overlap the RR of 1, thus indicated the intervention is better than the control [prophylactic effect of IgY: pooled RR, 0.38 (95% CI 0.27 to 0.55); therapeutic effect of IgY: pooled RR, 0.11 (95% CI 0.06 to 0.22) (Table 7 and Table 8)]. To assess the difference in the effect of IgY against type of diarrheal pathogen (bacterial or viral) 19 studies of bacterial pathogen and 5 studies of viral pathogen were considered (regardless of treatment strategy – therapeutic or prophylactic). The results suggested no statistical significance at the individual study level but the pooled effect measure showed the significant effect of IgY against both bacterial and viral diarrhea [Bacterial: pooled RR, 0.29 (95% CI 0.213 to 0.40; Viral: pooled RR, 0.45 (95% CI 0.28 to 0.70)]. Heterogeneity was considerably low at all the sub-group analysis.

**Table 7:** Prophylactic effect of IgY against diarrhea in Piglets.

Study	No. of mortality by diarrhea (or) No. with diarrhea/No. in Group (%)		Outcome measure considered - Mortality (M) or Diarrhea (D)	Relative Risk (RR)	95% Confidence Interval (CI)
	Intervention	Control			
**Animal Class: Pig**					
**Bacterial Pathogen**					
Imberechts 1997	2/6 (33)	4/6 (66)	D	0.500	0.141 to 1.772
	2/8 (25)	6/8 (75)	D	0.333	0.0941 to 1.181
	0/8 (0)	2/8 (25)	M	0.200	0.0112 to 3.576
Marquardt 1999	1/8(12.5)	5/8(26.5)	M	0.200	0.0296 to 1.351
	0/10(0)	3/10 (30)	M	0.143	0.00837 to 2.438
Owsu-Asiedu 2002	7/24(30)	13/18 (73)	D	0.404	0.203 to 0.802
	8/24(33)	18/18 (100)	D	0.333	0.189 to 0.587
Owsu-Asiedu 2003a	1/15(6.6)	6/15 (40)	M	0.167	0.0227 to 1.222
Owsu-Asiedu 2003b	0/18 (0)	8/24(33)	M	0.078	0.00480 to 1.265
Chernysheva 2004	8/12 (66)	8/12 (66)	D	1.000	0.568 to 1.761
Chu 2006	1/6 (16.7)	4/6 (66.7)	M	0.250	0.0383 to 1.633
Li 2009	0/4(0)	3/4 (75)	D	0.143	0.01000 to 2.041
Sarandan 2010	0/3 (0)	3/3 (100)	D	0.143	0.0110 to 1.860
**Viral Pathogen**					
Kweon 2000	5/19 (26)	10/17(58)	M	0.447	0.191 to 1.048
Zuo 2009	1/8 (12.5)	4/7 (57)	M	0.219	0.0314 to 1.526
Vega 2012	0/4(0)	6/6(100)	D	0.111	0.00814 to 1.516
**Pooled random effects**				**0.381**	**0.265 to 0.549**

**Test for Heterogeneity**: Q = 19.39; DF = 15; P = 0.1965, I^2^ = 22.65% (95% CI for I^2^ = 0.00 to 57.44).

**Table 8:** Therapeutic effect of IgY against Diarrhea in Piglets.

Study	No. of mortality by diarrhea (or) No. with diarrhea/No. in Group (%)		Outcome measure considered - Mortality (M) or Diarrhea (D)	Relative Risk (RR)	95% Confidence Interval (CI)
	Intervention	Control			
**Animal Class: Pig**					
**Bacterial Pathogen**					
Yokoyama 1992	0/7 (0)	6/7 (86)	M	0.077	0.00521 to 1.136
	0/4 (0)	4/4 (100)	M	0.111	0.00814 to 1.516
	0/5 (0)	4/5 (80)	M	0.111	0.00768 to 1.608
Xiao 1998	0/7 (0)	6/7 (85.7)	M	0.077	0.00521 to 1.136
	0/7 (0)	5/7 (71.4)	M	0.091	0.00603 to 1.370
Yang 2002	0/7 (0)	6/7 (85.7)	M	0.077	0.00521 to 1.136
	0/4 (0)	4/4 (100)	M	0.111	0.00814 to 1.516
	0/5 (0)	4/5 (80)	M	0.111	0.00768 to 1.608
Xu 2002	0/6 (0)	2/6 (33.3)	M	0.200	0.0117 to 3.406
	0/6 (0)	4/6 (66.7)	M	0.111	0.00738 to 1.673
	0/6 (0)	3/6 (50)	M	0.143	0.00907 to 2.249
Chu 2006	0/8 (0)	2/8 (25)	M	0.200	0.0112 to 3.576
**Viral Pathogen**					
Song 2003	1/6 (16.7)	6/6 (100)	M	0.167	0.0278 to 0.997
Cui 2012	0/10 (0)	10/10 (100)	M	0.048	0.00318 to 0.712
**Pooled random effects**				0.112	0.0558 to 0.223

**Test for Heterogeneity**: Q = 1.12; DF = 13; P = 1.0, I^2^ = 0% (95% CI for I^2^ = 0.00 to 0.00).

The pooled effect estimate in all the animal class suggested the beneficial effect of IgY against diarrhea, but majority of effect measure at the individual study level in animal class piglets and calves were not statistically significant at 95% confidence interval. All the studies selected for analysis indicated that, there was no adverse event because of the IgY or immune yolk administration either with feed or drinking water or force feeding. Some of the studies have analyzed the effect of probiotics along with IgY as an agent to enhance the beneficial bacterial population in the gut and decrease the pathogen count synergistically.

### 4. Publication bias

The presence of publication bias was primarily assessed by Funnel plotting. The asymmetry of funnel plots indicated the presence of publication bias in the study topic. Sometimes asymmetry could be the results of other sources rather than publication bias. In order to test asymmetry of funnel plots, Egger regression test was performed for included studies of each animal class separately. Results revealed that, there was some evidence of publication bias in the meta-analysis of animal class piglets and mice. But, it is difficult to reach any conclusion for meta-analysis of animal class poultry and calves, since the included studies are less in number (data not shown).

## Discussion

The prime findings of this SR and meta-analysis reveal that, the use of IgY as an alternative treatment approach could reduce the risk of diarrhea in domesticated animals with the statistically significant overall effect measure (pooled RR). The result was consistence across all the animal class including piglets, mice, poultry and calves. In addition, the 95% confidence intervals of majority of the studies in animal class mice and poultry did not embrace the RR of 1, thus indicates the statistical significance of IgY effect at individual study level. Despite, the animal class piglets and calves showed no evidence of the same effect at study level. Subgroup analyses using the studies reported in piglets also demonstrate the significant effect of IgY at meta-analysis level but failed to show the same evidence in individual studies. The genetic heterogeneity of animals might be the reason for differences in the statistical data between individual study level and overall effect measures of animal class piglets and calves. Overall outcome of the review shows that, the pooled RR of every analyses are significantly evidence the effect of IgY for reducing the deleterious events (morbidity or mortality) in treated animals when compare to the untreated control. This finding strengthens one of the promising approaches of antibody engineering, to apply IgY for treating and controlling the gastrointestinal infections in farm animal by oral passive immunization.

Diarrhea in young animals is one of the most important diseases that affect livestock industry and continuing to cause high economic losses worldwide due to increased mortality, medication costs, feed conversion rates and decreased weight gain. Normally these diseases are being controlled by administering antibiotics. Nevertheless, the problem of antibiotic resistance has increased dramatically during the past three decades; this may turn into successful treatment more difficult or sometimes impossible and prompted the ban on sub therapeutic use of antibiotics in livestock. Noticeably the sale of antibiotics used in veterinary medicine declined worldwide, for instance 16% of the decline in the sales from 2010 to 2011was reported by European surveillance of veterinary antimicrobial consumption, whereas the Swissmedic declared the fall of 8% in veterinary antibiotics sale in Switzerland during 2012 [74], [75]. Further, the development of novel antimicrobial agents has drastically declined, nevertheless only two new systemic classes of antibiotics (oxazolidinones and cyclic lipopeptides) and two topical classes of antibiotics (pseudomonic acids and pleuromutilins) were introduced in the market during the past 30 years [76]. As a result, the recent scientific publications are pointing out the need for next generation antibiotics. Therefore, new treatment approaches to substitute antibiotics are currently indispensable for combating pathogens. At this juncture, the beneficial effect of IgY likely to be considered for further investigation as a viable alternative to antibiotics. In addition to the bacterial and viral diarrheal diseases in live stock, some economically important intestinal diseases are caused by several different protozoan species; for instance, avian coccidiosis is caused by Eimeria protozoa in the poultry worldwide. In recent years, IgY has also been focused as an alternative control method for the prevention and treatment of protozoan diseases by oral passive immune therapy. Reports on this approach are also clearly evidence the significant protective effect of IgY against *Eimeria tenella* and *Eimeria maxima* in chicks. This provide a strong suggestion, that the passive immunization using IgY could be extended to many other gastro intestinal tract diseases caused by microorganisms irrespective of their types and groups [77]–[79].

In recent decades, a number of IgY or hyper immune egg products are available in the market for the betterment of overall health in humans, livestock and companion animals. As a result, many biological companies are now focusing their attention to establish IgY production, especially for animal feed supplementation. Results of this SR is relatively supports those commercial products unless otherwise manufactured with scientific approach.

Regarding the study quality and risk of bias, none of the studies were scored 100% as per ARRIVE Guidelines. Of course it was not possible for the earlier reports to have contained the standard protocol for animal experiment, but some of the recent studies also did not fit the standards. Only very few recent reports have strictly followed the proper guidelines for the intervention of therapeutic events in animals. The risk of bias assessment showed, that majority of the items had unclear risk of bias out of 10 items (considered for the study). These findings exhibit the inadequacy of reporting or designing animal experiments in scientific publications. Further, it enforces the need and importance of proper animal experiments in future pre-clinical trials to reduce the bias by outlining transparency in objective and methodology.

The methodological heterogeneity among the included studies, especially the dose and formulation of IgY (purified form or immune yolk/egg) was fairly considerable. With respect to reduction of the possible heterogeneity between the studies, the binary outcome such as the number of animals that died (mortality) due to diarrhea and the number of animals that exhibited diarrhea during and after treatment with IgY were considered as primary outcome measures for the meta-analysis. However, the two animal class (pigs and calves) and subgroup analyses showed statistical significance in overall random effect, but not at individual study level. This would have possibly influenced by several factor including the poor animal experimental design, the difference in the effect of IgY between primary outcomes measures taken into consideration for this SR and other continuous variables, the divergence in biological response of each animal class towards a particular treatment strategy.

In terms of limitations to this SR, the inclusion of four different animal classes in a single study has driven the reviewers to consider some homologous binary outcome measures between the studies. Some other continuous variables with respect to pathogenesis of diarrhea such as fecal consistency, number of bacteria or viral particle excreted per g of feces, differences in the body weight gain were not included in the meta-analysis. As a consequence, the reliability of the conclusion (the pooled effect size) of this meta-analysis is relatively low. This limitation is also applicable for subgroup analysis. The further crucial scrutiny by separate meta-analysis of each animal class with the focus to analyze multiple outcome measures is warranted.

In conclusion, the present SR and meta-analysis demonstrated the beneficial effect of IgY in controlling and preventing the diarrhea in domesticated animals. This supports the opinion that IgY is useful for prophylaxis and treatment of gastrointestinal infection by oral passive immunization as an alternative strategy to antibiotics. However, the further investigations are indispensable for the robustness of IgY application are indispensable to optimize the effective IgY dose with suitable formulation in order to withstand in the gastric environment; to determine the combinatorial effect of IgY with other alternative therapeutic strategies including probiotics and plant extracts for the improved performance. The gold standard animal experiments are necessarily to be conducted as per guidelines.

## Supporting Information

Table S1 Characteristic of the included studies – Piglets.
(DOC)

Table S2 Characteristic of the included studies – Mice.
(DOC)

Table S3 Characteristic of the included studies – Poultry.
(DOC)

Checklist S1 PRISMA Checklist for SR and Meta-analysis.
(DOC)

Protocol S1 Systematic Review protocol (publicly available in CAMARADES site).
(DOC)
