# Supplementary material for: Effect of Chicken Egg Yolk Antibodies (IgY) against Diarrhea in Domesticated Animals: A Systematic Review and Meta-Analysis
Source: PLoS One. 2014 May 20;9(5):e97716. doi: 10.1371/journal.pone.0097716 (PMC4028221; doi:10.1371/journal.pone.0097716)
Supplement: Table S1 — Characteristic of the included studies – Piglets. (DOC) [file pone.0097716.s001.doc]

**Additional File 1: Characteristic of the included studies - Piglets**

| **Author & Year** | **Experimental Animal** | **Challenge Dose** | **IgY Treatment** | **Outcome Assessment**  **(Type of Efficacy)** |
| --- | --- | --- | --- | --- |
| **Animal Class: Piglets**  **Bacterial Pathogen** | | | | |
| **Yokoyama et al., 1992** | Colostrums deprived Piglets (4 hours after birth) | Trial 1 – K88^+^ ETEC 10^12^ CFU  Trail 2 – K99^+^ ETEC 10^12^ CFU  Trail 3 – 987p^+^ ETEC 10^10^ CFU | 4mL of Anti- K88, -K99^+^, -987p antibody solutions – 3 times/day for 3 consecutive days after the occurrence of diarrhea [Titer 156, 625 and 2,500 for treatment groups respectively in 3 Trials] | Fecal consistency, weight loss, rectal swabs and mortality rate (T) |
| **Kellner et al., 1994** | Piglets | Field trail – Piglets with diarrhea were selected and grouped | 3gm of egg yolk powder in 5mL of water once/day by oral deliver | Morbidity, duration of diarrhea and fecal consistency were observed (F) |
| **Erhard et al., 1996** | Piglets | Field trail – Piglets with diarrhea were screened and grouped | Whole egg powder (contains specific antibody against *E. coli* K88, K99, 987P and rotaviurs) 5% was added to the standard feed. | Mortality, duration of diarrhea and fecal consistency was observed |
| **Imberechts et al., 1997** | Weaned Pigs – 3 to 4 weeks of age | Exp1:10^9^ CFU of  *E. coli* 107/86 edema disease strain  Exp2:10^9^ CFU of  Post weaning diarrhea *E. coli* strain 571 | Exp1: 30g of immune egg powder/day/pig for 11 days (before 16 h to challenge)  Exp2: All things similar as Exp1 except infected with  *E. coli* 571 and egg yolk powder was given for 12 days | Excretion of bacteria was examined by culture technique (P) |
| **Yokoyama et al., 1997** | 4 weeks old large white pigs | 10^11^ CFU of viable *E. coli* /animal daily for three days | 1:10 and 1:50 titer premixed with feed ration (for 9 days) | Fecal consistency, intestinal swabs (C) |
| **Zuniga et al., 1997** | Swiss Landrace secondary SPF Pigs – 23-30 days | Exp1: *E. coli* strain 3064/84 – 6.7 log CFU/mL  Exp 2: *E. coli* strain 8199 6.7 lof CFU/mL  F18 Positive ETEC-  In both the experiment  Challenged during experimental days 11-13 and 30-32 | Exp1 High egg antibody – Basal feed +5% undiluted Egg powder from immunized hens low egg antibody – Basal feed + 1% immune egg powder and 4% non-immune egg powder  Egg powder mixed with feed and given from experimental day 10 – 29 | Rectal swabs at 1-2 days interval (P) |
| **Xiao et al., 1998** | Piglet – 4 hours old | Exp1:1.0X10^12^CFU/mL of E. coli [C83901-K88]/animal  Exp2:1.0X10^12^CFU/mL of E. coli [C83917] 987P/animal | Exp1:After challenge 4mL of anti-K88, [1:625, 1:250titer] yolk antibody given 3 times/day for 3 day  Exp2:After challenge 4mL of anti-987p Ciliac protein [1:625, 1:250titer] yolk antibody given 3 times/day for 3 day | Observed the onset of diarrheal symptoms and mortality rate (Sherman et al.,) (T) |
| **Marquardt et al., 1999** | Exp1: Cotswold piglets – 3day old  Exp2: Cotswold piglets – 21day old  Filed Trail: 14-18days old piglets | Exp1: 5mL of  *E. coli* K88+ MB – 10^10^ CFU/mL per piglet at time 0 h  Exp2:5mL of  *E. coli* K88+ MB – 10^12^ CFU/mL/piglet at time 0 h and 5h  Filed trail | Exp1:  1.5g of egg-yolk antibodies at a titer of 140000 -3 times on day first [-3, 0 and 3 hours] and then 1time/day for 2 consecutive day  Exp2: 0.5g of egg-yolk antibodies at a titer of 140000 -3 times a day [-1, 4 and 9 hours] for 2 consecutive day  Filed trail :  Fed with a diet containing egg yolk antibodies including 2% anti-K88+ and 0.1% anti-K99+ antibodies and 1.7% SDEY with no antibodies  Piglets fed with the three experimental diets for 8 days. | Occurrence of diarrhea, fecal consistency, weight loss and mortality (P and F) |
| **Yang et al., 2002** | Piglet – 4 hours old | Exp1: 10^12^CFU/mL of ETEC K88^+^  Exp2: 10^12^CFU/mL of ETEC K99^+^  Exp3: 10^10^CFU/mL of ETEC 987P+ | Water soluble fraction of IgY powder with different titer (1:156, 1:625, 1: 2500) – 3times/day for 3 days | Fecal score and mortality were observed (T) |
| **Xu et al., 2002** | Piglets (one day old) | Exp1:K88  Exp2:K99  Exp3:987P  1.0X10^10^CFU/mL of ETEC per animal at 24 hours of age | After onset of diarrhea the piglets received 2mL of IgY solution 3times/day for 7 days | Fecal index according to Sherman et al., and mortality rate were assessed (T) |
| **Owsu-Asiedu et al., 2002** | Weaned Piglets at 10day of age | 6mL of *E. coli* (ETEC) suspension [10^10^ CFU/mL] on day 7 | 0.5% with feed [0.2 and 0.3% of anti-K88 or anti-F18 IgY antibodies] | Severity of diarrhea by fecal consistency (P) |
| **Owsu-Asiedu et al., 2003a** | 17 day old pig early-weaned pigs | On experimental day 7 - 10^10^ CFU/mL concentration 6mL/pig | 0.3% and 0.2% egg yolk powder [0.5%] containing specific anti-K88 and F18 antibodies respectively | Severity of diarrhea by fecal consistency and mortality (P) |
| **Owsu-Asiedu et al., 2003b** | 17 day old pig early-weaned pigs | On experimental day 7 - 10^10^ CFU/mL concentration 6mL/pig | 0.3% and 0.2% egg yolk powder [0.5%] containing specific anti-K88 and F18 antibodies respectively | Severity of diarrhea and mortality (P) |
| **Chernysheva et al., 2004** | Pigs – 3 to 4 weeks of age | 5mL of a suspension *E. coli* (K88+) [ETEC] [10^11^ CFU/mL] / pig  Challenged on the third day | Treat1 – 3.2g/kg of egg-yolk antibody powder or 3.2% of the total diet [recommended by the manufacturer]  Treat2 – egg yolk antibody powder was added 10 times of recommended level [32g/kg or 32% of the total diet] | Prevalence of diarrhea and mortality (P) |
| **Girard et al., 2006** | Weaned Pigs – 17days old | 1mL of 1.0x10^10^ CFU of PEPC strain ECL1001 Nal^r^ in 9mL of TSB on Day6, 7, 8 and 9 | 3g of spray-dried egg powder – containing approximately 637mg protein/mL of anti-intimin IgY/pig/day from day 4 until necropsy. | Presence of diarrhea, dehydration, food and water intake (P) |
| **Chu, et al., 2006** | Exp1:Piglet 3days old  Exp2: Piglet 21 days old  Filed trail: Piglet 10-20 days old - 40 | Exp1: 5mL of 1.0X10^10^CFU/mL of ETEC per animal at age of 3days  Exp2: 5mL of 0.2X10^12^CFU/mL of ETEC per animal at age of 21days after 5 hours the same dose given to each animal again  Field Trail: Infected animals with E. coli diarrhea by diagnosis | Exp1: 3hours before challenge with ETEC – 5g of egg yolk powder was given- then after 3 hours of challenge and for 4 days in 5mL of normal saline  Exp2: After 1^st^ challenge with ETEC 5g of egg yolk powder was given at 1, 4^th^ and 8th (3 hours after second challenge) hours of experiment and continued the same for 4 days.  5mL containing1g/ 3g /5g of egg yolk powder per day.  Field Trail: 5mL solution containing1g/ 3g /5g of egg yolk powder per day by oral administration | Observed Fecal consistency according to Sherman et al., and mortality rate (P, T and F) |
| **Li et al., 2009** | Belgian Landrace Pigs – 40day old | 5mL of *E. coli* (K88+) [10^11^CFU/mL] /pig at time 0 hour | Treat 1: 0.4g of non-encapsulated IgY  Treat 2: 2g of microencapsulated IgY  (-1, 4, and 9 h after challenge) | Occurrence of diarrhea, fecal score, weight gain & recovery (P) |
| **Sarandan et al., 2010** | Piglet – 7day old | 1mL of *E.coli* after 24hrs of IgY product administration then after 12 hrs- 2mL of *E. coli* [10^10^ bacteria/mL] | IgY product [Globigen, Ghen Corporation, Japan] – 2mL at 40 minutes interval until the end of the experiment | Histopathological study after 77h of post challenge (P) |
| **Liou et al., 2011** | LYD piglets – 21 days old | 5mL of *E.coli* K99 at a dose of 2x10^10^CFU/mL/piglet 2times/day for 2days. Administered in 0 and 6 hours of the experiment | 2g of egg yolk antibodies [65.28µg/mL Water soluble fraction] 2 times/day for 2 consecutive days after 1^st^ challenge. | Occurrence of diarrhea, fecal consistency and mortality (C) |
| **Viral Pathogen** | | | | |
| **Kweon et al., 2000** | 3days old piglets | 5LD_50_/mL dose of wild Porcine Epidemic Diarrhea Virus (PEDV) | Animal Experiment: 2-3mL of IgY three times for one day before challenge and throughout the exp. after challenge  Filed Application: piglet showed with diarrhea in two or three days of age received 2-4mL of IgY twice/thrice per day for a week | Clinical signs and Mortality (P & F) |
| **Song et al., 2003** | Piglets (one day old) | 5times of minimum dose need to cause infection – PEDV-Oral route/animal | 2mL of IgY solution -3times/day/animal for 5 days | Determined fecal consistency(index) according to Sherman et al., and mortality rate (T) |
| **Zuo et al., 2009** | New born Seghers Piglets – 3days old | Prophylactic efficacy: 5LD_50_ TGEV/piglet  Therapeutic efficacy:  Ppig farms having outbreak of diarrhea [TGEV], part of the piglets from the same litters were used for the study | Prophylactic efficacy: 3mL of IgY [64N] 3times/day before challenge exposure.  After challenge the oral administration of IgY continued throughout the experiment  Therapeutic efficacy:  3mL of IgY 2times/day for a week | Clinical signs and Mortality (P & F) |
| **Cui et al., 2012** | Piglet-1day old | 2mL/day of PEDV and PTGV 3 times/day/animal | 2mL of egg yolk antibody 2times/day/animal by oral route | Incidence of diarrhea and Death were estimated (T) |
| **Vega et al., 2012** | Gnotobiotic pigs 24hours of age | 10^6.7^ FFU of HRV/5mL | 10mL of HRV IgY 1024 / HRV IgY 4096 / VP6 IgY 4096/ control IgY/HRV IgG 4096 in 210 of sterile Ab free milk at 24 hours age until day 12. | Fecal consistency, virus shedding by ELISA, IFA etc. (P) |

**Legend:** CFU colony forming unit, ETEC Enterotoxigenic *E. coli*, PEPC Porcine Enteropathogenic *E. coli,* TGEV Porcine transmissible gastroenteritis virus, HRV Human Rotavirus, Type of Efficacy: P-Prophylactic Effect; T-Therapeutic Effect; F-Field Trial; C-Simultaneous challenge and treatment.
