# Supplementary material for: Effect of Chicken Egg Yolk Antibodies (IgY) against Diarrhea in Domesticated Animals: A Systematic Review and Meta-Analysis
Source: PLoS One. 2014 May 20;9(5):e97716. doi: 10.1371/journal.pone.0097716 (PMC4028221; doi:10.1371/journal.pone.0097716)
Supplement: Table S2 — Characteristic of the included studies – Mice. (DOC) [file pone.0097716.s002.doc]

**Table S2: Characteristic of the included studies - Mice**

| **Author & Year** | **Experimental Animal** | | **Infection Dose** | | **IgY Treatment** | **Outcome Assessment**  **(Type of Efficacy)** |
| --- | --- | --- | --- | --- | --- | --- |
| **Animal Class: Mice**  **Bacterial Pathogen** | | | | | | |
| **Peralta et al., 1994** | Mice 5-7 weeks old (25g) | 0.2 mL of SEF 14 [2 x 10^10^ CFU] pretreated of 0.2mL of egg yolk antibody solution from immunized egg [titer 32 and 128] and non-immune egg [titer <10] separately | | Egg yolk antibody solution 0.2mL/mouse by gastric intubation with a blunt needle and repeated 3 times/day for 3 days | | Clinical signs of infection and survival rate (P) |
| **Yokoyama et al., 1998** | BALB/c SPF Mice – 4 weeks old | Trial 1: 0.2mL of *Salmonella enteritidis* [1x10^10^ CFU/mL]  Trial 2: 0.2mL of S. *typhimurium* [1x10^8^ CFU/mL]  by gastric intubation | | Trial 1: 0.2mL of antibody solution containing of anti-OMP [1:256], anti-LPS [1:128], anti-Fla[1:1024]were given after 30minutes of challenge and 3 times/day for 3 consecutive days  Trial 2: 0.2mL of antibody solution containing of anti-OMP[1:256], anti-LPS[1:128], anti-Fla[1:2048]were given after 30minutes of challenge and 3 times/day for 3 consecutive days | | Clinical signs of infection and survival rate (C) |
| **Jacoby, 2001** | Mice 25-30gm | 0.3mL of Castor oil on day 2 after treatment with egg powder | | 20mL/kg distilled water – spray dried immune egg powder contain antibody against *E. coli, Shigella, Staphylococcus and Salmonella* for two consecutive days | | Presence of diarrhea by stool assessment either normal or soft or loose stool (T) |
| **Wang et al., 2010** | BALB/c female mice – 15 to 17g | 5 times LD_50_ of Stx1.  Enterohemorrahagic *Escherichia coli* O157:H7 | | IgY 0.2, 0.4, 1.2, 3.6 mg was incubated with Stx1 prior to injection at 4^o^C overnight | | Survival time of the mice was observed for 7 days (P) |
| **Hirai et al., 2010** | 4 day old suckling mice | *V. cholerae* (VC) O139 and O1 – 7.5x10^8^ CFU/mL. 50µL of each cell suspension given at 4 hours after the mice were separated from mother by intragastrically. | | For therapeutic study: 50µL of each IgY [1mg/mL] – anti-VC, anti-CT B or both given 3 hours after challenge and returned to their mother. Surviving mice repeatedly administered with the same IgY for up to 72 hours with different intervals [every 2, 4, 6 or 12h]  For prophylactic study: 2mg, 1mg and 0.5mg/mL were mixed with *V. cholerae* O1 or O139 , 50 µL of the mixture orally inoculated | | Survival time of the mice was observed (P&C) |
| **Neri et al., 2011** | ddY Mice [4weeks of age] | Mortality assay: 625µg/kg of partially purified Stx-1 [2.5 times LD_50_] 17.4µg/kg of partially purified Stx-2 [2 times LD_50_] Mouse survival was observed for 10 days  Protective efficacy: Mice challenged with i.p. injection of ppStx-1 or ppStx-2 | | Mortality assay: 2.5mg, 10mg, 25mg and 100mg/kg of Anti-Stx-1 IgY or Anti-Stx-2 IgY  Protective efficacy:  i.v. injection with 50mg and 100mg/kg of Anti-Stx-1 IgY or Anti-Stx-2 IgY | | Mortality rate was observed at different time intervals of IgY treatment (T) |
| **Parma et al., 2011** | NIH Mice of 10-14g | 5LD_50_ Wild type Shiga toxin 2 holotoxin [pre-incubated with Two-fold dilutions of IgY and IgG diluted in PBS | | 0.07µg/mL IgG and 0.55µg/mL IgG [Rabbit] or  0.55 µg/mL IgY and 8.75 µg/mL IgY [Chicken] – 0.5mL each. | | Symptoms of disease and death recorded for days (T) |
| **Feng et al., 2013** | Female Kunming Mice [6 weeks old] | 0.3mL of Shiga toxin2e [839µg/mL] 6 hours later IgY administration | | 0.3mL of Anti-Shiga toxin2e IgY  Gp1: No dilution; Gp2: 1:2 Gp3: 1:4 and 0.3mL of Anti-Shiga toxin2 e B protein IgY Gp4: No dilution, Gp5: 1:2, Gp6: 1:4  Given by intraperitoneal injection | | Symptoms and death monitored for 5 days (T) |
| **Viral pathogen** | | | | | | |
| **Bartz et al., 1980** | Mice (3days old) | Epidemic diarrhea of infant mice virus >5000 mouse infectious dose/10µL-30minutes after 1^st^ dose of IgY | | Water-soluble fraction 10µL twice/day (for four days) IgY (30days after immunization) | | Examined dilatation, inflammation in the upper intestine and presence of liquid yellow contents in the rectum (C) |
| **Hatta et al., 1993** | Suckling mice BALB/c Mice [6 days old] | 50µL of 3.5 x 10^7^ FCFU of HRV Mo strain/mouse | | 50µL of Anti-HRV (Mo) IgY solution (225µg) /mice at the desired time before or after HRV inoculation | | Inspected for diarrhea by gentle palpation of the abdomen –incidence of diarrhea after 48h challenge (C) |
| **Kuroki et al., 1993** | Suckling Mice – 7 day old | Bovine Rota Virus:  25µL/mouse – 3.55 DD_50_ (10^7.5^ TCID_50_) for Shimane strain and 3.98 DD50 (10^7.0^ TCID_50_) for KK-3 strain. | | Homotypic or Heterotypic IgY treatment:  25µL of each dilution of IgY against Shimane and KK-3  For Shimane Challenge:  Anti-Shimane – 640, 160, 40 NAT  Anti-KK-3 – 10240, 2560 NAT  For KK-3 Challenge:  Anti-KK-3 – 2560, 640, 160, 40 NAT Anti-Shimane – 10240, 2560 NAT (3hrs before challenge) | | The onset of diarrhea was observed clinically from 24 to96 hours post challenge (P) |
| **Sarker et al., 2007** | Four day old pups [Balb/c mice] | 2x10^7^ foci-forming units of rhesus rotavirus in a 10µL volume on 0^th^ day | | IgY in total volume of 10µL PBS commenced from day 1 – 5.  Four different doses: 10mg/mL; 1mg/mL; 0.1mg/mL and 0.01mg/mL | | Prevalence and duration of diarrhea (T) |
| **Ngyuen et al.,** | BALB/cMice 6 to 8 weeks of age | 250 pfu (5xLD50) of A/PR/8/34 (H1N1) virus,  1,000 pfu (5xLD50) of A/Philippines (H3N2), 10xLD50 of VN/  1203 (H5N1) or 5x LD50 A/Aquatic bird/Korea/W81/2005  (H5N2) resuspended in 50µL PBS per animal | | Treated by intranasal route with 50µL of IgY before or after infection | | Mortality rate was measured (C) |
| **Liou et al.,** | 1 day old ICR strain mouse | 1x10^5^ pfu of Enterovirus71 strain MP4 –Mouse adapted 4643 strain of Enterovirus71  Trail 7:3x10^6^ pfu of EV71 strain MP4/mouse | | Trail 1-4:100µL of IgY /mouse with different neutralization titers (64, 128, 256, and 512) was injected IP into the Challenged mice 1-3 days after inoculation (dpi-day of post infection) for 3 consecutive days.  Trail 5&6: IgY treatment dates were 2-4dpi and 4-6dpi respectively.  Trail 7: Oral treatment of IgY – mice 4-5 days old  Trail 8: Oral treatment of IgY – mice 5-6 days old (titer 512 – 1h before or 1h after challenge) | | Morbidity and Mortality was measured for 2 weeks after challenge (P &T) |
| **Buragohain et al., 2012** | BALB/c mouse – 4/5days old | 4000 EEP of HRV-3/mice | | Exp1: 12 hours post inoculation of virus - 50µL anti-HRV-3IgY/animal [3 different doses – 1.25, 0.625, 0.3125mg/mL] twice/day for 3 days  Exp2: Pre-exposure treatment- 50µL [250 and 125 µg/mL] of anti-HRV-3IgY in two groups separately 2hours prior to oral challenge with 2000EEP of HRV-3 | | Prevalence, severity and duration of diarrhea (P & T) |

**Legend:** CFU colony forming unit, SPF Specific pathogen free, OMP Outer membranous protein, Fla Flagellin, LPS Lipopolysaccharide, Stx Shigatoxin, LD Lethal dose, FCFU Fluorescent cell forming unit, TCID Tissue culture infective dose, HRV Human Rotavirus, Type of Efficacy: P-Prophylactic Effect; T-Therapeutic Effect; F-Field Trial; C-Simultaneous challenge and Treatment
