# Supplementary material for: Effect of Chicken Egg Yolk Antibodies (IgY) against Diarrhea in Domesticated Animals: A Systematic Review and Meta-Analysis
Source: PLoS One. 2014 May 20;9(5):e97716. doi: 10.1371/journal.pone.0097716 (PMC4028221; doi:10.1371/journal.pone.0097716)
Supplement: Table S3 — Characteristic of the included studies – Poultry. (DOC) [file pone.0097716.s003.doc]

**Table S3: Characteristics of the included studies - Poultry**

| **Author & Year** | **Experimental Animal** | **Challenge Dose** | **IgY Treatment** | **Outcome Assessment**  **(Type of Efficacy)** | |
| --- | --- | --- | --- | --- | --- |
| **Animal Class: Poultry**  **Bacterial Pathogen** | | | | | |
| **Tsubokura et al., 1997** | Chicken  Exp1: Prophylaxis – 14 days old; Exp2:Therapy – 18 days old [after 4 days from initial infection] | Exp1:10^6^ *Campylobacter jejuni* pre-incubated with 0.5g IgY  Exp2: NA | Exp1: 0.5g/Chicken  Exp2: 0.2g/Chicken (95% purity) | | Bacterial culture was performed before and after treatment (P & T) |
| **Fulton et al., 2002** | One day old commercial female Pekin ducklings | *Salmonella enteritidis* phage type 9B  Exp1: 0.5ml of 1.4 x 10^6^ CFU /duckling  on Day 1 (Gp1) and Day 5 (Gp2)  Exp2: 0.5ml of 2.04 x 10^7^ CFU/duckling Day1 (Gp1) and Day5 (Gp2) | Exp 1: 15ml of IgY (ELISA titer: 94,800) mixed per 3.84 L of drinking water from Day 1  Exp 2:  IgY Gp: Antibody 2ml/duckling in 7.68L of water in the morning  IgY+ Probiotic Gp: Antibody 2ml/duckling in 7.68L of water in the morning and 0.1ml probiotics (pro) /duckling in their evening drinking water | | Monitored for clinical signs of illness, euthanized and necropsies and culture were performed (P & T) |
| **Gurtler et al, 2004** | White leghorn SPF Layer Hens – 47 weeks (Exp1) and 52 weeks (Exp2) of age | *Salmonella enteritidis* (SE)  Exp1: 2x10^9^ CFU/hen  Exp2: 2x10^8^ CFU/hen | Both Exp. Egg powder started before 5 days from infection  Exp1: 3g/day/hen with commercial feed till 18^th^ day of post infection  Exp2: 3g/day/hen with commercial feed till 21^st^ day of post infection | | Presence of bacteria in the eggs was determined by culture method (P) |
| **Kariyawasam et al., 2004** | Chickens – 11 days old | 0.2 ml of a bacterial suspension  Exp 1: Homologous *E.* *coli* O78 EC99 (10^7^ CFU)  Exp 2: *E. coli*  O2 (107 CFU)  Exp 3: *E. coli* O1 (10^4^ CFU)  Exp 4: *E. coli* O78 EC99 but birds pre-exposed to Infectious bronchitis virus (IBV) | Chickens in IgY treatment groups were injected intramuscularly  with 100 mg of respective IgY against some selected E. coli antigens ( anti-live *E. coli,* anti-killed *E. coli* , anti-PapG, anti-IutA , anti-LPS, and a mixture of  anti-FimH, anti-PapG, anti-IutA and anti-LPS) in 1ml of PBS by intramuscular route | | Mortality and macroscopic lesions were observed (P) |
| **Adriana, 2007** | Quails – 70 days of age | *Salmonella gallinarum*  Trail 1: 10^8^CFU/ml/bird  Trail 2:  10^7^CFU/ml/bird | IgY 1ml/bird for 14 days | | Eggs were analyzed for *S. gallinarum* (P &T) |
| **Rahimi et al., 2007** | 1 day old Ross 308 Broiler chicks | 1x 10^8^CFU/ml/bird at 3 day of age | 15ml of yolk per 3ml drinking water on day 1 after challenge and till end of the experiment | | Bacterial culture was done and calculated protection factor (T) |
| **Chalghoumi, et al., 2009** | 1 day old male Ross 308 broiler chicks | *Salmonella* spp. mix (2 × 10^6^ cfu/mL per  chick) – 4 day old | 5% of feed (w/w) egg yolk powder with 5 different ratio hyper immune yolk powder (100:0, 75:25, 50:50, 25:75, 0:100) – from day 1 | | Infection factor and protection factor were calculated as per the method of Mead *et al.,* 1989 (P) |
| **Tamilarasan et al., 2009** | 1 Day old boiler chicks | 10^9^ cfu of *E.coli* (O:2) *S. pullorum*, *Clostridium perfringens* and *C. jejuni* respectively for 3 consecutive days. | 3 ml of IgY against *E. coli*, *S. pullorum, C. jejuni* and *C. perfringens* | | Necropsy, histopathology and bacteriological examination (P) |
| **Mahdavi et a., 2010** | 1 day old Boiler chicks (Ross:308) | 0.5 mL of *E. coli* O78:K80 at day 7 and then for additional 7 consecutive  days from day 14 to 21 with 1.0 mL of a late log  phase culture (10^9^ cfu/mL) | 0.1, 0.2, or 0.4% (wt/wt) specific IgY (sIgY) from eggs of immunized hens or with  0.2 or 0.4% (wt/wt) nonspecific IgY powder (nsIgY) in diets | | Intestinal *E. coli* enumeration by culture technique, serological and immunological tests (T) |
| **Wu et al., 2013** | Chicken – 3days old | Prophylactic effect:1X10^8^CFU of *S. pullorum*/0.2ml per bird by IM after IgY administration  Therapeutic effect: 1X10^8^CFU of *S. pullorum*/0.2ml per bird by intramuscular | 10, 20, 30,40g of IgY powder mixed with basal feed – given 3 days before challenging  40, 50, 60 and 70g IgY powder mixed with basal feed after challenging for 3 days | | FCR and mortality were calculated |
| **Viral Pathogen** | | | | | |
| **Muhammad et al., 2001** | Field trail - Broiler chicks at farm | Broiler farm with problem of IBD - Infected birds were grouped | Diluted yolk containg  16, 32, 64, 128 units of anti-IBDV antibody titer | | Birds were examined for mortality for 10 days (F) |
| **Malik et al., 2006** | Field trail -Chicken – mixed breeds in infected farm | Farm with outbreak of IBD - Diseased birds were grouped | Agar gel precipitation units [AGPT] of 256, 128, 64, 32 were given to group 1, 2, 3 and 4 | | Birds were examined for mortality for 10 days (F) |
| **Rahimi et al., 2007** | Day old specific pathogen free (SPF) white Lohmann chickens | 0.1ml of liver virus with 10^7^ egg-infectious dose (EDI50)/ml on day 22 intranasally | 15ml of antibody mixed per 3.84L of drinking water – from day 17 until end of the experiment | | Virus shedding investigated by egg inoculation and hemagglutination assay (P) |
| **Abd El-Ghany 2011** | 1 day old Hubbard broiler chicks | Virulent IBDV strain - 10^5^ EID50/bird through eye drop installation method on day 35 | Gp1: Live IBDV vaccine at 7 and 14 days old  Gp:2 0.5ml/bird -IgY in PBS at day 28  Gp:3 Live IBDV vaccine on Day 7 and 14 days & IgY-day 28 | | Clinical signs, Morbidity, Mortality – Dead birds examined for IBDV gross lesions. Burs/BW ratio – Histopathology (P) |
| **Farooq et al., 2012** | 14 days old Golden birds | IBDV - 50% w/v bursal homogenate – 100, 200 and 100µl by ocular, nasal and cloacal route respectively | Exp 1: Gp 1 , 2, 3 treated with 0.1ml of IgY at 8000, 6000, 4000 titer of IgY respectively – subcutaneous route | | Signs and Symptoms – Postmortem examination – Morbidity & Mortality |

**Legend:** CFU colony forming unit, IBV Infectious bronchitis virus, Type of Efficacy: P-Prophylactic Effect; T-Therapeutic Effect; F-Field Trial.
