# Supplementary material for: Effect of Chicken Egg Yolk Antibodies (IgY) against Diarrhea in Domesticated Animals: A Systematic Review and Meta-Analysis
Source: PLoS One. 2014 May 20;9(5):e97716. doi: 10.1371/journal.pone.0097716 (PMC4028221; doi:10.1371/journal.pone.0097716)
Supplement: Protocol S1 — Systematic Review protocol (publicly available in CAMARADES site). (DOC) [file pone.0097716.s005.doc]

**Protocol S4: Systematic Review protocol (publicly available in CAMARADES site)** (http://www.camarades.info/index_files/Protocols.html).

**Effect of Chicken Egg Yolk Antibodies (IgY) against Diarrhea in Domesticated Animals: A Systematic Review and Meta-analysis**

Thirumalai Diraviyam^1^, Bin Zhao^1, 2^, Yuan Wang^1^, Ruediger Schade^3^, Antonysamy Michael^4^, and Xiaoying Zhang^1*^

^1^College of Veterinary Medicine, Northwest A&F University, Yangling 712100, P.R. China

^2^ College of Science, Northwest A&F University, Yangling 712100, P.R. China

^3^ Institute of Pharmacology, Charité - Universitätsmedizin Berlin, Germany

^4^PSG College of Arts and Science, Bharathiar University, Coimbatore 641014, Tamil Nadu, India

**^*^ Corresponding author:**

Tel./Fax:+86 29 8709 1239. E-mail *address*: zhang.xy@nwsuaf.edu.cn (Dr. X.-Y. Zhang)

**Abstract:** Internationally many researchers have reported on the beneficial effect of IgY oral passive immune-therapy against various bacterial and viral pathogens as a promising alternative strategy to the conventional treatment. However, the results of experimental applications of IgY have not always been consistent and make the application of IgY to commercial use a difficult task. Hence, this systematic review was focused to evaluate the effect of IgY against diarrhea in different animals classes. This protocol summarizes the steps used in this systematic and meta-analytic review. Previous research reports were retrieved from the different electronic database, and then the potential literatures were identified on the basis of criteria for inclusion or exclusion. Quality of the included studies was assessed using ARRIVE guideline and they were subjected for risk of bias assessment. Then the data were extracted from the potential studies and analyzed systematically with meta-analysis. This review could be a platform for the further research and applications of IgY as therapeutic tool against diarrhea in animals. In addition, this will be a timely contribution for the researchers those who are focusing on IgY as an alternative therapeutic tool to antibiotics.

***Key words:*** IgY meta-analysis, Diarrhea, Piglets, Chicken, Mice, Calves, Passive immunization

**1. Introduction**

A major milestone in man’s progress from primitive existence to civilized life is settled agriculture and domestication of livestock [1]. Disease prevention and low mortality are the greatest challenge from the ancient practice to the current modern livestock management. In a common way antibiotics have been used in animal agriculture for growth promotion (sub-therapeutic doses), disease prevention (prophylactic doses) and for the treatment (therapeutic dose) for more than 50 years [2] and many of the research report and practical experience have shown the usage of antibiotic significantly contribute for the improved performance of animals [3]. However, the use and misuse of in-feed antibiotics have resulted in serious complications due to drug residues in animal products and increased bacterial resistance. The recognition of these dangers prompted the ban on sub therapeutic usage of antibiotics in many developed countries and some of the developing countries are seriously considering a similar ban. Therefore a viable alternative strategy to antibiotics is essentially needed to combat drug-resistance microorganisms and also to treat the diseases that are unresponsive to drug therapy (viral infection) and for individuals with impaired immune systems who are unable to respond to conventional vaccines [4].

A broad range of products as an effective alternative to antibiotics have been/are being focused seriously by the global biomedical research community. Recently, passive immunization using chicken egg yolk immunoglobulin (IgY) has become an attractive approach with considerable attention as it possesses a variety of advantages over mammalian IgG such as convenience, high yield and cost-effectiveness. Oral administration of specific chicken IgY has been shown to be effective against a variety of intestinal pathogens especially diarrheal pathogens in different animal classes and humans such as bovine and human rotaviruses, bovine coronavirus, enterotoxigenic *Escherichia coli* (ETEC) and *Salmonella* spp. [5]. Although the beneficial effects of chicken IgY in controlling or preventing the Diarrheal disease in animals have been known for more than two decades and reported by many researchers globally, it still remains a difficult task to use chicken IgY as an alternative to conventional treatment with strong scientific conclusion. At this juncture, a meta-analysis is necessary and helpful to summarize the previous research findings and provide a comprehensive conclusion for therapeutic use of chicken egg yolk antibodies as well as proper direction for further research.

**2. Objectives**

1. To collect the articles from different databases (MEDLINE, EMBASE, SPRINGER-LINK, WILEY, AGRICOLA, MEDWELL Journals, Scientific Publish) regardless of language.
2. To cover the major animal classes (Pigs, Ruminants, Mice and Poultry (chicken) already focused by research community to apply IgY as therapeutic tool.
3. To consolidate the research outputs from different studies and analysis them statistically to make comprehensive conclusion.
4. Presenting our comparison as systematic review (meta-analysis) for the reference of the researchers to conduct experiment on IgY against Diarrhea

**3. Methods**

***3.1. Literature search:***  Previous research reports were retrieved from different electronic data bases such as MEDLINE, EMBASE, SPRINGER-LINK, WILEY, AGRICOLA, MEDWELL Journals, Chinese articles from Core periodicals in 2012, Scientific Research Publish regardless of language and publication status. Bibliographies of narrative review articles and eligible trials were reviewed manually for the potential studies which do not retrieved by the electronic searches. Besides, the publications in the conference proceedings, dissertation abstracts and other studies that would have to be in “file drawers” (unpublished manuscripts) were also considered for the study in order to reduce the risk of missing potential studies. The following search strategy has been used for PubMed and then slightly modified for other databases. The same key words were translated into German and Chinese for searching German and Chinese articles.

***Search Strategy***:

| 1. IgY antibodies | 1. Chicken IgY |
| --- | --- |
| 1. Chicken Egg Yolk Antibodies | 1. IgY therapy |
| 1. IgY prophylaxis | 1. IgY passive immune therapy |
| 1. Chicken antibodies against diarrhea | 1. Chicken IgY against diarrhea |
| 1. IgY therapy and diarrhea | 1. IgY passive protection and diarrhea |
| 1. IgY treatment and broiler chicks | 1. IgY#Diarrhea#calves |
| 1. IgY#Diarrhea#mice | 1. Chicken IgY#Diarrhea#piglets |
| 1. IgY and Broiler Chickens | 1. IgY and pigs |

***3.2. Selection of potential studies:*** Research reports that were published in English, Chinese and German were selected and included for the analyses if they met following inclusion criteria: 1) In-vivo animal experiment was conducted according to the laws and regulations of animal ethical committee 2) Study should be evaluated the effect of IgY against diarrhea causing pathogens 3) Study should be used any one formulation of IgY: purified IgY, partially purified IgY, immune yolk (liquid) or immune yolk powder (spray dried or freeze dried). 4) The treatment effect of IgY was assessed by any of the following observations: mortality rate, survival rate, fecal score for pathogen excretion, fecal consistency (diarrhea) or body weight. 5) Article must contain sufficient data for extraction such as number of animals in each group (treatment and control), challenge dose and treatment dose with duration, study hypothesis to evaluate either prophylactic or therapeutic effect of IgY.

***3.4. Data Extraction:*** Outcome of the each included potential studies were extracted using a standardized data extraction form (prepared by ourselves). Missing information was discussed with the corresponding authors by contacting through e-mail or telephonic conversation. Reviewers assessed the risk of bias, inconsistency and indirectness of the study included for the systematic review. List of information extracted from the each study as follows,

1. Author and year of publication (to access the development of research chronologically)
2. Purpose of the study (either therapeutic or prophylactic use of IgY)
3. Type of trial (experimental or field)
4. Experimental animal class (pigs, mice, poultry, ruminants), type of breed, age, body weight, numbers used and grouping system
5. Source of diarrhea (experimental or natural), if experimental type of pathogen (bacterial or viral), strain, challenge dose, duration, time and mode of administration
6. IgY treatment protocol including the formulation (purified IgY, partially purified IgY, immune yolk or whole immune egg – powder or liquid), dose, time and mode of administration
7. Method of outcome assessment, results of experimental group and control groups
8. Conclusion of the study ( supporting the IgY treatment or not) and reference analysis (to identify the missing of published study by electronic search)

***3.5. Assessing the quality of the Studies:*** Potential studies were assessed for methodological quality using the Animal Research: Reporting of In-vivo Experiment (ARRIVE) Guidelines developed as a part of NC3Rs (National Centre for the Replacement, Refinement and Reduction of Animals in Research) to improve the design, analysis and reporting of research using animals [6, 7]. The assessment was considered the key information in the included articles as per guideline for giving score. Maximum score was 20 points per article, according to the score; articles were categorized in to three different groups as good, moderate and poor if the score percentage was ≥75%, ≥65% and ≤55% respectively. Reviewers assessed the risk of bias, inconsistency and indirectness of the study included for the systematic review.

***3.6. Data Analysis:*** In accordance with the differences between studies such as different doses, formulation of IgY, pathogenic strains used for challenge, duration of treatment, outcome assessment and age of animals; the random-effects meta-analysis was taken in to consideration for calculating the relative risk (RR) and the 95% confidence interval (CI) in trails reporting the binary outcome (as primary outcome measure) i.e., incidence of diarrhea or mortality. To estimate the amount of heterogeneity *I*^2^ value was calculated [8]. Other study characteristics were summarized narratively with tables and running text.

**4. Outcome and Conclusion (Under Progress)**

The therapeutic or prophylactic effect of IgY against diarrhea in farm animals is the main concern of this systematic review. With that focus the studies are being intensively compared and analyzed statistically. A comprehensive conclusion will be drawn based on the result outcome and consolidated evidence will be presented on the use of IgY for the prevention and control of treatment. A list of recommendation for future studies in this area will be summarized.

**Contact details for further information**

Prof. Dr. Xiao-Ying Zhang,

Post Box 19, College of Veterinary Medicine, Northwest A&F University (North Campus), Xinong Rd 22, Yangling, Shaanxi Province, 712100, China

Tel & Fax: +86 29 8709 1239; E-mail: [zhang.xy@nwsuaf.edu.cn](mailto:zhang.xy@nwsuaf.edu.cn)

| **Anticipated or actual start date**  1st April 2013 | **Anticipated Completion date**  30th November 2013 |
| --- | --- |

**Funding sources/sponsors:**

This work was supported by the Research Fellowship for International Young Scientists (No. 31350110512), China National Science Foundation, the International S&T Cooperation Project of Northwest A&F University (2013), Key Program for International S&T Cooperation Projects of Shaanxi Province (2012KW-20) and Ministry of Education and State
Administration of Foreign Experts Affairs "Overseas Teacher" project (No.MS2011XBNL057), China.

**Conflicts of interest:** None Known

**Date of Protocol Registration in CAMARADES –** 15th November 2013

| **Stage of review at time of this protocol submission** | **Started** | **Completed** |
| --- | --- | --- |
| Literature search in electronic data base | - | Yes |
| Study selection process | - | Yes |
| Screening according to eligibility criteria | - | Yes |
| Data extraction | - | Yes |
| Quality Assessment | - | Yes |
| Data analysis | Yes | In progress |

**Reference**

1. Singh YP, Naresh RK, Sing PK, Singh HL, Rathi RC, Sing SV. Crop – livestock interaction to food security and sustainable development of agriculture for small and marginal farmers in northwest India. The Journal of Rural and Agricultural Research 2011; 11(2): 6-13.
2. Turner JL, Dritz SS, Minton JE. Review: alternatives to conventional antimicrobials in swine diets. Professional Animal Scientist. 2001; 17:217–26.
3. Cromwell GL. Why and how antibiotics are used in swine production. Animal Biotechnology 2002; 13:7–27.
4. Kovacs-Nolan J, and Mine Y. Egg Yolk Antibodies for Passive Immunity. Annual Review of Food Science and Technology 2012; 3:163-182.
5. Xu Y, Li X, Jin L, Zhen Y, Lu Y, Lu, Li Y. Application of chicken egg yolk immunoglobulins in the control of terrestrial and aquatic animal diseases: A review. Biotechnology Advances 2011; 29: 860-868.
6. Kilkenny C, Browne W, Cuthill IC, Emerson M, Altman DG. Animal research: reporting in vivo experiments: the ARRIVE guidelines. British Journal of Pharmacology 2010; 160: 1577–1579.
7. Hooijmans CR, Ritskes-Hoitinga M. Progress in Using Systematic Reviews of Animal Studies to Improve Translational Research. PLOS Medicine 2013; 10(7): e1001482. doi:10.1371/journal.pmed.1001482.
8. Vesterinen HM, Sena ES, Egan KJ, Hirst TC, Churolov L, Currie GL, Antonic A, Howells DW, Macleod MR. Meta-analysis of data from animal studies: A practical guide. Journal of Neruoscience Methods 2014; 221:92-102.
